# Latent TGF-β Activation Is a Hallmark of the Tenascin Family

**DOI:** 10.3389/fimmu.2021.613438

**Published:** 2021-05-13

**Authors:** Alexandre Aubert, Perrine Mercier-Gouy, Stéphanie Aguero, Laurent Berthier, Sophie Liot, Laura Prigent, Lindsay B. Alcaraz, Bernard Verrier, Raphaël Terreux, Catherine Moali, Elise Lambert, Ulrich Valcourt

**Affiliations:** ^1^ Laboratoire de Biologie Tissulaire et Ingénierie Thérapeutique (LBTI), UMR CNRS 5305, Université Lyon 1, Institut de Biologie et Chimie des Protéines, Lyon, France; ^2^ Institut de Recherche en Cancérologie de Montpellier (IRCM), INSERM U1194, Université de Montpellier, Institut du Cancer de Montpellier (ICM), Montpellier, France

**Keywords:** tenascins, transforming growth factor (TGF)-β, latent TGF-β activation, tissue homeostasis, tumor microenvironment, immune cell modulation

## Abstract

Transforming growth factor-β (TGF-β) isoforms are secreted as inactive complexes formed through non-covalent interactions between bioactive TGF-β entities and their N-terminal pro-domains called latency-associated peptides (LAP). Extracellular activation of latent TGF-β within this complex is a crucial step in the regulation of TGF-β activity for tissue homeostasis and immune cell function. We previously showed that the matrix glycoprotein Tenascin-X (TN-X) interacted with the small latent TGF-β complex and triggered the activation of the latent cytokine into a bioactive TGF-β. This activation most likely occurs through a conformational change within the latent TGF-β complex and requires the C-terminal fibrinogen-like (FBG) domain of the glycoprotein. As the FBG-like domain is highly conserved among the Tenascin family members, we hypothesized that Tenascin-C (TN-C), Tenascin-R (TN-R) and Tenascin-W (TN-W) might share with TN-X the ability to regulate TGF-β bioavailability through their C-terminal domain. Here, we demonstrate that purified recombinant full-length Tenascins associate with the small latent TGF-β complex through their FBG-like domains. This association promotes activation of the latent cytokine and subsequent TGF-β cell responses in mammary epithelial cells, such as cytostasis and epithelial-to-mesenchymal transition (EMT). Considering the pleiotropic role of TGF-β in numerous physiological and pathological contexts, our data indicate a novel common function for the Tenascin family in the regulation of tissue homeostasis under healthy and pathological conditions.

## Introduction

The Tenascin family is a group of large extracellular matrix (ECM) glycoproteins composed of four members, Tenascin-C (TN-C), Tenascin-R (TN-R), Tenascin-W (TN-W, also known as Tenascin-N) and Tenascin-X (TN-X), sharing a common modular structure. Tenascins are composed of heptad repeats at the amino terminus, followed by several Epidermal Growth Factor (EGF)-like domains, different numbers of Fibronectin type III (FNIII) repeats and a C-terminal globular domain resembling Fibrinogen (FBG-like domain) (1). The amino-terminal cysteine-rich region with heptad repeats, forming the Tenascin assembly domain, allows individual subunits to oligomerize into disulfide-linked trimers, permitting TN-R and TN-X to form structures called “tri-brachions” (2, 3). TN-C and TN-W are able to form hexamers, also known as “hexa-brachions”, due to the presence of an additional cysteine residue within this domain forming an extra disulfide bridge between two trimers (4, 5).

Tenascin family members have very specific expression patterns. TN-R (160-180 kDa), which is restricted to the nervous system, is expressed by oligodendrocytes and subtypes of neurons in the central nervous system and by Schwann cells in the peripheral nervous system. TN-R plays a major role in the regulation of neural stem cell progenitor proliferation and differentiation (6), and its expression is upregulated during axonal myelinization (7). Mutations found in the TN-R encoding gene (*TNR*) are associated with predisposition for neurodevelopmental pathologies, such as attention deficit hyperactivity disorder (8) and non-progressive form of spastic disorders (9). TN-X (450 kDa), encoded by the *TNXB* gene, is the largest member of the Tenascin family and is constitutively present in adult connective tissues, including tendons, ligaments, digestive tract and dermis. TN-X interacts with fibrillar (types I, III and V) collagens, fibril-associated (types XII and XIV) collagens and other matrix components (Decorin) (3, 10), and is believed to regulate the spacing between collagen fibrils (11). Through this architectural function, TN-X might provide the connective tissue with appropriate biomechanical properties to support tissue and organ functions (12). Consequently, TN-X deficiency causes classical-like Ehlers-Danlos Syndrome (EDS) in Human (13), a rare and hereditary connective tissue disorder characterized by generalized joint hypermobility, skin hyperlaxity and easy bruising (14). TN-C (220 kDa) and TN-W (180 kDa), which are mainly expressed during embryonic development, remain barely detectable in adult tissues, with a very restricted expression in several stem cell niches. Indeed, TN-C has been found in neural and epithelial stem cell niches, whereas TN-W has been associated with the osteogenic one (15).

Nevertheless, TN-C and TN-W levels are often up-regulated under physio-pathological stresses, including fibrosis, angiogenesis, wound healing and tumor progression (16–18). Indeed, TN-C and TN-W are often *de novo* expressed in different types of cancer, in which they display well characterized pro-tumoral activities by promoting tumor cell proliferation (19), cell migration and invasiveness (20), and metastasis formation (21). Consequently, these two glycoproteins have been proposed as biomarkers for most solid tumors (*i.e.* breast, colorectal and pancreatic cancers) (22, 23), in which TN-C has been often associated with poor clinical outcome (22, 24). While far less studied, TN-R and TN-X have also been identified as being involved in tumor progression. Due to its specific localization, TN-R expression has been tightly related to brain cancer development. Indeed, TN-R was described as down-regulated in glioblastomas, medulloblastomas, ependymomas and meningiomas, while up-regulated in gangliogliomas, suggesting that its role as either a tumor-promoting or tumor-suppressing factor is highly context-dependent (25, 26). In contrast to TN-C and TN-W, we recently demonstrated that (*i*) TN-X is down-regulated at both mRNA and protein levels in the six cancers with the highest incidence and mortality worldwide (*i.e.* lung, breast, colorectal, prostate, stomach and liver cancers) and (*ii*) a low level of *TNXB* transcripts is correlated with a worse overall survival in patients suffering from breast or lung cancers (27). Even if the molecular mechanisms involved are still unknown, these observations suggest that TN-X might exert tumor-suppressive properties.

In addition to its architectural function within the ECM, we also identified a novel role for the TN-X in the regulation of cell signaling. Thanks to its C-terminal FBG-like domain, TN-X was found to regulate Transforming Growth factor (TGF)-β bioavailability (28). The three TGF-β isoforms (TGF-β1, 2 and 3) are pleiotropic cytokines secreted by a broad range of cell types in an inactive form. Indeed, they are synthesized as pro-proteins and form disulfide-linked homodimers that are proteolytically processed before secretion. Upon cleavage, the pro-domain, called latency-associated peptide (LAP), remains non-covalently bound to the mature (bioactive) TGF-β moiety, maintaining it in a latent state by preventing its exposure to cell-surface receptors. Latent TGF-β can be found as a soluble entity, called the small latent complex (SLC), but also as a large latent complex (LLC), in which the SLC is covalently bound to a Latent TGF-β Binding Proteins (LTBP), thus maintaining a reservoir of latent TGF-β into the matrix (29). In order to exert its physiological roles, latent TGF-β has to be activated, meaning that the mature TGF-β entity has to be released from the SLC. Depending on the cell and/or tissue context, latent TGF-β activation may result from (*i*) a proteolytic cleavage within the LAP pro-domain and the subsequent release of the mature TGF-β and/or (*ii*) a conformational change in the LAP, allowing exposure of the TGF-β entity (30). During the latter process, mature TGF-β remains in interaction with its LAP pro-domain, but the bioactive sites of the cytokine are unmasked and able to bind to TGF-β type II receptor (TβRII) at the cell surface (31). Activated TβRII then recruits and activates the TGF-β type I receptor (TβRI) by trans-phosphorylation. In the canonical TGF-β signaling pathway, TβRI phosphorylates Smad2 and Smad3, allowing them to interact with Smad4 in order to enter the nucleus and act as transcription factors that regulate a large set of TGF-β-responsive genes (32). These genes are involved in key biological mechanisms, such as embryonic development, tissue homeostasis and repair, through stem cell fate orientation, cell cycle arrest (cytostasis), epithelial-to-mesenchymal transition (EMT), cell motility and immuno-modulation. TGF-β cytokines also play a dual role during cancer development, acting either as oncogenic or tumor-suppressing factors depending on the cellular context (32, 33).

Based on the conserved modular structure between the four members of the Tenascin family, we speculated that TN-C, TN-R and TN-W share with TN-X the ability to regulate latent TGF-β activation through their C-terminal globular domain. Indeed, amino-acid sequence alignment revealed that FBG-like domains of the four Tenascins are highly conserved, sign of a potential functional redundancy. Herein, we demonstrated that full-length Tenascins, and their respective FBG-like domains, are produced and purified in association with SLC components (*i.e.* TGF-β1 and its LAP pro-domain). Interestingly, recombinant FBG-like domains, presented to cells in either immobilized or soluble form, have the ability to induce the canonical TGF-β/Smad intracellular pathway in epithelial cells, suggesting that the molecular association between the C-terminal part of the Tenascins and latent TGF-β promotes the activation of the cytokine. Consequently, epithelial cells cultured onto immobilized FBG-like domains underwent cell cycle arrest (cytostasis) and partial EMT, two cellular programs induced by active TGF-β. Altogether, our results indicate that latent TGF-β activation is a common feature of the Tenascin family, providing new insights into the mechanisms by which these glycoproteins may regulate tissue homeostasis under healthy and pathological conditions.

## Materials and Methods

### Cell Culture and Reagents

Normal Murine Mammary Gland (NMuMG) epithelial cells were obtained from ATCC (American Type Culture Collection) and cultured in complete Dulbecco modified Eagle’s medium (DMEM), containing 10% (v/v) Fetal Bovine Serum (FBS, Gibco), 1% (v/v) Penicillin-Streptomycin (PS, Gibco), and supplemented with 10 μg/mL Insulin (Roche). Human Embryonic Kidney (HEK)-293 cells expressing Epstein-Barr Nuclear Antigen 1 (EBNA1) were purchased from Invitrogen and maintained in the same complete medium in the presence of 250 μg/mL of G418 (Geneticin, PAA) as recommended by the manufacturer.

HEK-293 EBNA1 cells stably transfected with pCEP4-BM40-6HIS/Puromycin encoding full-length human TN-C and TN-W, respectively donated by G. Orend (U1109 INSERM, Strasbourg, France) and R. Chiquet-Ehrismann (Friedrich Miescher Institute for Biomedical Research, Basel, Switzerland), were cultured in complete DMEM containing 2.5 µg/mL of Puromycin (Gibco). HEK-293 cells stably transfected with pSecTag2/hygromycin expression vector (Invitrogen) encoding either full-length bovine TN-X (from Gly^23^ to Gly^4,135^ residues according to the GenBank reference NM_174703) or its TN-X^ΔEΔF^ derivative (consisting of FNIII modules only, from Gly^745^ to Thr^3,910^ residues) (3, 34), were cultured in complete DMEM containing 400 µg/mL of Hygromycin B (Thermo Fischer Scientific).

Recombinant human TGF-β1 was purchased from PeproTech and dissolved in 4mM HCl containing 0.1% (w/v) Bovine Serum Albumin (BSA, Euromedex) for cell culture experiments. For neutralization experiments, mouse monoclonal IgG1 anti-TGF-β1/β2/β3 (Clone 1D11) antibody was purchased from R&D Systems and isotype-matched mouse Gamma Globulin used as negative control from Jackson ImmunoResearch. In both cases, cells were incubated for 10 min at 37°C in presence of 5 or 10 μg/mL of either anti-pan-TGF-β or isotype-matched antibody before stimulation.

### Plasmids, Cloning and Cell Transfection

For recombinant protein production in mammalian cells, FBG-like sequences of human TN-X (from Gly^4,048^ to Gly^4,272^ residues according to GenBank ID 7148), TN-C (from Gly^1,338^ to Ala^1,564^; GenBank ID 3371), TN-W (from Val^990^ to Phe^1,229^; GenBank ID 63923) and TN-R (from Gly^1,027^ to Phe^1,257^; GenBank ID 7143) were cloned between NheI and BamHI restriction sites into the pCEP4-BM40-8HIS/Puromycin expression plasmid (gift from M. Koch, University of Cologne, Germany) using the One-Step Sequence- and Ligation- independent Cloning (SLIC) method (35) and specific oligonucleotides (**Table 1**). cDNAs encoding the FBG-like domains of human TN-C and TN-W were amplified using full-length protein-encoding plasmids (36) as templates. Prior to SLIC cloning, the cDNA encoding the FBG-like domain of human TN-R was amplified from human brain mRNA (Clontech) by RT-PCR using the PrimeStar HS DNA Polymerase (Takara Bio Inc.) and the following forward 5’-GGAGGCCGGGTGTTCCCTCATC-3’ and reverse 5’-CTCAGAACTGTAAGGACTGCCG-3’ oligonucleotides. Similarly, the cDNA encoding the FBG-like domain of TN-X was obtained by RT-PCR from human primary fibroblast mRNA using the following forward 5’-GGTGGGCTGCGGATCCCCTTC-3’ and reverse 5’-GCCTCCCCCCGCTGGGGAGC-3’ primers. The integrity of the cloned sequences was assessed by direct sequencing (Eurofins Genomics).

**Table 1:** Oligonucleotide primers used for the cloning of the FBG-like domains in the pCEP4-BM40-8HIS/Puromycin vector using the SLIC method.

Primer name	Primer Sequence
pCEP4-hFBGC-Forward	5’-**CTTTGCCTGGCCGGGAGGGCTCTGGCAGCCCC** GCTAGCAGGACTCCTGTACCCCTTCCCC-3’
pCEP4-hFBGC-Reverse	5’-**GGATCATTAATGGTGGTGATGATGGTGGTGGTG** GGATCCTGCCCGTTTGCGCCTGCCTTC-3’
pCEP4-hFBGR-Forward	5’-**CTTTGCCTGGCCGGGAGGGCTCTGGCAGCCCC** GCTAGCAGGAGGCCGGGTGTTCCCTCAT-3’
pCEP4-hFBGR-Reverse	5’-**GGATCATTAATGGTGGTGATGATGGTGGTGGTG** GGATCCGAACTGTAAGGACTGCCGTTT-3’
pCEP4-hFBGW-Forward	5’-**CTTTGCCTGGCCGGGAGGGCTCTGGCAGCCCC** GCTAGCAGTTGGTGCCCGTTTCCCACAC-3’
pCEP4-hFBGW-Reverse	5’-**GGATCATTAATGGTGGTGATGATGGTGGTGGTG** GGATCCGAACGTTCGCAGCCTTCCTCT-3’
pCEP4-hFBGX-Forward	5’-**CTTTGCCTGGCCGGGAGGGCTCTGGCAGCCCC** GCTAGCAGGTGGGCTGCGGATCCCCTTC-3’
pCEP4-hFBGX-Reverse	**5’-TGGATCATTAATGGTGGTGATGATGGTGGTGGTG** GGATCCGCCTCCCCCCGCTGGGGAGC-3’

Vector tail sequences are indicated by bold face. Restriction sites are underlined.

A cleavage site attributed to the Furin-like family of pro-protein convertases was identified at the C-terminal sequence of human FBG-W domain, thus allowing the removal of the histidine tag during biochemical purification of the recombinant protein. Consequently, site-directed mutagenesis was performed on the FBG-W-encoding pCEP4-BM40-8HIS plasmid to substitute the Arg^1,220^ amino-acid by an alanine residue, using the QuikChange II XL Site-Directed Mutagenesis Kit (Agilent Technologies) and the following forward 5’-TGTCCTGGGCAGAAAGAAGGCGACGCTGAGAGGAAGGCTG-3’ and reverse 5’-CAGCCTTCCTCTCAGCGTCGCCTTCTTTCTGCCCAGGACA-3’ oligonucleotides. The recombinant FBG-W^RKKA^ domain was used throughout the study.

HEK293-EBNA1 cells at 70-90% confluency in 6-well plate were transfected with 4 μg of pCEP4-BM40-8HIS/Puromycin plasmid encoding FBG-like domain using Lipofectamine 2000 (Invitrogen), in serum- and antibiotic-free DMEM for 48 hours. Mock-transfected cells, corresponding to HEK-293 EBNA1 cells transfected with empty pCEP4-BM40-8HIS/Puromycin vector were used as a negative control. Once transfected, the G418 antibiotic was replaced by 2.5 μg/mL of Puromycin (Gibco) in the culture medium.

For recombinant protein production in bacteria, cDNAs encoding FBG-like domains of the four human Tenascins were amplified by PCR (Taq Platinum HF polymerase, TermoFisher), flanked with appropriate restriction sites (**Table 2**) and ligated into the linearized and dephosphorylated pT7.7 vector (United States Biochemical Corp.) using Rapid DNA ligation kit (Roche). Integrity of the cloned sequences was assessed by direct sequencing (Eurofins Genomics). Chemically ultracompetent *E.coli* BL21-DE (3) bacteria (New England Biolabs) were transformed using heat-chock method and selected on LB-agar plates containing 100 μg/mL ampicillin (Sigma-Aldrich).

**Table 2:** Oligonucleotide primers used for the cloning of the FBG-like domains in the pT7.7 vector using standard method.

Primer name	Primer sequence	Restriction site
pT7-7-hFBGC-Forward	5’-CGCGGATCCGGACTCCTGTACCCCTTCCCC-3’	*BamHI*
pT7-7-hFBGC-Reverse	5’-CCCCTGCAGTGCCCGTTTGCGCCTGCC-3’	*PstI*
pT7-7-hFBGR-Forward	5’-GCTAGAATTCGCGGAGGCCGGGTGTTCCCT-3’	*EcoRI*
pT7-7-hFBGR-Reverse	5’-CCCCTGCAGGAACTGTAAGGACTGCCGTTT-3’	*PstI*
pT7-7-hFBGW-Forward	5’-CGCGGATCCGTTGGTGCCCGTTTCCCACAC-3’	*BamHI*
pT7-7-hFBGW-Reverse	5’-CCCGTCGACGAACGTTCGCAGCCTTCC-3	*SalI*
pT7-7-hFBGX-Forward	5’-GCTAGAATTCGCGGTGGGCTGCGGATCCCC-3’	*EcoRI*
pT7-7-hFBGX-Reverse	5’-CCCCTGCAGGCCTCCCCCCGCTGGGGAGCG-3’	*PstI*

Restriction sites are underlined.

### Recombinant Protein Production, Purification and Adsorption

For protein production in mammalian cells, stably transfected HEK-293 or HEK-293 EBNA1 cells were cultured over confluence for 3 weeks under serum deprivation and culture medium was collected 3 times a week. Conditioned media enriched in recombinant proteins or collected from mock-transfected cells were cleared from cellular debris by centrifugation for 10min at 400g at 4°C and stored at -80°C.

Full-length TN-X and TN-X^ΔEΔF^ were purified by mean of two chromatographic steps as described previously (3). Briefly, a first affinity chromatography was performed on Heparin-Sepharose column (GE Healthcare). After two washes with 50 mM Tris-HCl, pH 8.0, proteins were eluted using 0.5 M NaCl in 50 mM Tris-HCl, pH 8.0, dialyzed 3 times against 50 mM Tris-HCl pH 8.0 (twice for 2 hours and once overnight) and applied on a Q-Sepharose column (GE Healthcare). Elution was performed using linear NaCl gradient (from 0 M to 1 M) in 50 mM Tris-HCl, pH 8.0 and fractions enriched in recombinant proteins were pooled.

Full-length TN-C and TN-W were purified as described by Giblin et al. (2018) (37). Before purification, histidine-tagged full-length TN-C and TN-W were precipitated from conditioned medium by stirring 2h at 4°C in presence of 2.2 M ammonium sulfate (Sigma-Aldrich), pelleted by centrifugation at 12,000g, for 20 min at 4°C, resuspended in 50 mL of Phosphate-Buffered Saline (PBS, Euromedex) containing 0.01% Tween-20 (VWR) and dialyzed 3 times (twice for 2h and once overnight) at 4°C in PBS containing 0.01% Tween-20. In order to remove Fibronectin (FN), a first gelatin-Agarose column (Sigma-Aldrich) was performed, in which FN stayed attached to the gelatin beads while TN-C or TN-W passed to the flow-through. This flow-through containing TN-C or TN-W was then applied on a Ni-Nitriloacetic acid agarose (Ni-NTA, Qiagen) column, which was subsequently washed twice using PBS containing 20 mM imidazole. TN-C or TN-W were then eluted using 300 mM imidazole diluted in PBS.

Prior purification, mock-conditioned medium or conditioned media enriched in human FBG-X, FBG-C, FBG-W or FBG-R were dialyzed using MWCO 6,000-8,000 Da dialysis tubing (SpectrumLabs) in 300 mM NaCl, 50 mM NaH_2_PO_4_, 5 mM imidazole (Sigma-Aldrich), overnight at 4°C. Thanks to the presence of eight histidine residues at the C-terminal end of recombinant FBG-like domains, a single Ni-NTA affinity column was performed. Column was washed twice using 300 mM NaCl, 50 mM NaH_2_PO_4_, 5 mM imidazole, and proteins were eluted with 200 mM imidazole (diluted in the washing buffer).

After an initial overnight dialysis against PBS, fractions enriched in recombinant proteins were dialyzed overnight against sterile PBS containing 0.2% (v/v) chloroform (Sigma-Aldrich) to prevent contamination, followed by a last overnight dialysis against sterile PBS before storage at -80°C. The homogeneity of recombinant proteins was assessed by SDS-PAGE coupled with R250 Coomassie Blue staining (Biorad), and protein concentration was determined using the QuantiPro™ BCA Assay kit (Sigma-Aldrich).

Recombinant CUB1CUB2 (C1C2) fragment of human Procollagen C-proteinase enhancer-1 (PCPE-1) containing eight histidine residues at its C-terminal end was produced in HEK-293 T cells, as previously described (38).

Recombinant proteins were diluted in PBS and adsorbed onto cell culture dishes or coverslips overnight at 4°C. When full-length Tenascins were used within the same experiment, the quantity of coated recombinant protein was 22.2 pmol/cm^2^, while FBG-like domains were coated either at 333 pmol/cm^2^ (corresponding to 10 μg/cm^2^), 666 pmol/cm^2^ (20 μg/cm^2^) or 999 pmol/cm^2^ (30 μg/cm^2^), depending on the experiments. Following passive protein adsorption, non-specific interaction sites were saturated using 1% (w/v) BSA diluted in PBS for 1h at 37°C.

For protein production in bacteria, transformed BL21-DE (3) cells were cultured in LB medium containing 100 μg/mL ampicillin until the 600nm absorbance was comprised between 0.6 and 0.8. Protein production was then induced by 1 mM Isopropyl β-D-1-thiogalactopyranoside (IPTG, Sigma) for 3h at 37°C. Bacteria were pelleted by centrifugation at 5,000g for 20min and insoluble FBG-like domains were extracted from inclusion bodies by stirring 1h at room temperature with 100 mM CAPS (Sigma) pH 11.0 containing 8 M urea (Promega). Protein lysate was cleared from bacteria debris by centrifugation at 5,000g for 20min and supernatant containing solubilized proteins were incubated for 1h at room temperature with Ni-NTA beads. Once loaded onto an empty column, histidine-tagged FBG-like domains bound to the Ni-NTA beads were washed and refolded using a linear decreasing urea gradient (concentration from 8 M to 0 M) diluted in 100 mM CAPS, each time using 10 column volumes. Elution was performed using 200 mM imidazole diluted in 100 mM CAPS, pH 8.0, and dialyzed twice overnight against sterile PBS at 4°C before storage at -80°C. Purity and concentration of recombinant proteins produced in bacteria was assessed as described earlier for proteins produced in mammalian cells. Recombinant FBG-like domains produced in bacteria were noted FBG* to distinguish them with those produced in mammalian cells.

The level of LPS in each recombinant protein preparation was assessed using the *Limulus amaebocyte* lysate assay (Pierce LAL Chromogenic Endotoxin Quantitation Kit) according to the manufacturer’s instructions. The levels of LPS in recombinant proteins purified from mammalian cell conditioned medium were below 50 pg/mL, whereas they were ranging between 100 and 500 pg/mL in the FBG-like domains purified from bacteria.

### Immunoblotting

For cell signaling analyses, either 10^6^ NMuMG cells were seeded and cultured for 3h in the presence of immobilized recombinant proteins or 5.10^5^ cells were seeded on plastic dishes, and stimulated the day after with soluble recombinant FBG-like domains for 1h prior to cell lysis. Total proteins were extracted from stimulated NMuMG cells using radioimmunoprecipitation assay (RIPA) extraction buffer (Thermo Scientific) containing EDTA-free protease inhibitor and phosphatase inhibitor cocktails (Roche) for 20min on ice. After centrifugation at 13,200g for 10min at 4°C, supernatant containing solubilized proteins were stored at -20°C prior to Western blot analyses. 20 µg of total proteins were resolved by SDS-PAGE in Tris-Glycine buffer containing Sodium-Dodecyl-Sulfate (TG-SDS, Euromedex) and then transferred onto Polyvinylidene Fluoride membrane (PVDF, Milipore) at 0.4 A during 2h in TG-SDS buffer containing 20% Ethanol. Membrane was saturated using 5 or 10% (w/v) non-fat dry milk diluted in Tris-buffered saline (TBS, Euromedex) containing 0.1% (v/v) Tween-20 (T-TBS, VWR) for 1h at room temperature prior overnight incubation with primary antibody (at 4°C). Rabbit monoclonal antibody anti-phospho-Smad2 (Ser465/467, diluted at 1/1,000 in T-TBS containing 5% (w/v) BSA) and rabbit polyclonal antibody anti-Smad2/3 (diluted at 1/500 in T-TBS containing 5% (w/v) BSA) were purchased from Cell Signaling Technology, goat polyclonal antibody anti-human LAP (TGF-β1) was purchased from R&D System (diluted at 1/100 in T-TBS containing 5% (w/v) non-fat dry milk), rat monoclonal antibody anti-TGF-β1 (diluted at 1/500 in T-TBS containing 5% (w/v) non-fat dry milk) was purchased from BD Pharmigen™, mouse monoclonal anti-6X His tag (diluted at 1/1,000 in T-TBS) was purchased from Abcam, monoclonal mouse antibody anti-GAPDH (diluted at 1/1,000 in T-TBS) was purchased from Abcam, and rabbit antibody anti-actin (diluted at 1/1,000 in T-TBS) was purchased from Sigma-Aldrich. Secondary anti-mouse IgG (Abcam, diluted at 1/10,000 in T-TBS containing 5% (w/v) non-fat dry milk), anti-rabbit IgG (Abcam, diluted at 1/5,000 or 1/2,500 in T-TBS containing 5% (w/v) non-fat dry milk), anti-goat IgG (ImmunoResearch Laboratories, diluted at 1/2,000 in T-TBS containing 10% (w/v) non-fat dry milk) and anti-rat IgG (ImmunoResearch Laboratories, diluted at 1/2,000 in T-TBS containing 10% (w/v) non-fat dry milk) antibodies coupled with Horseradish Peroxidase (HRP) were added for 1h at room temperature prior to signal detection using the enhanced chemiluminescence technique with the ECL™ prime Western Blotting Detection Reagent (GE Healthcare) and the FX Fusion CCD camera (Vilber Lourmat). Signals were quantified using FIJI software (SciJava).

### Cell Reporter Assay

100,000 NMuMG cells were seeded on 24-well culture plates on day 1. On day 2, cells were co-transfected with the pGL3-basic reporter plasmid (Promega) encoding firefly luciferase under control of TGF-β-responsive elements ((CAGA)_12_-Luc) (39) together with the pRL-CMV vector (Promega) encoding Renilla luciferase under the control of the cytomegalovirus ubiquitous promoter to determine the transfection efficiency and to normalize firefly luciferase activity. Transfection was carried out for 4h to 6h using lipofectamine 2000 (Invitrogen) according to manufacturer recommendations, using a 20:1 plasmid ratio corresponding to 0.8 μg of pGL3-MLP-(CAGA)_12_ and 0.04 μg of pRL-CMV per condition. Cells were then cultured in complete medium and stimulated for 16h using the amount of proteins described in figure legends. On day 3, cell lysates were obtained using Passive Lysis Buffer (Promega) for 15min at room temperature under shaking and were then kept frozen at -20°C. Luciferase assay was performed in Costar 96-well flat bottom white polystyrol plates using the Dual Glo luciferase reporter assay system (Promega) according to the recommendations of the manufacturer. Briefly, luciferase activity was measured for 7s per well using 20 μL of cell lysate and 100 μL of LAR II buffer. Then, luciferase activity was quenched and Renilla was activated by adding 100 μL of Stop&Glo solution per well, followed by a reading of Renilla activity for 7 seconds. Luminescence measurements were performed on Tecan i-control Infinite M1000 (Tecan Group).

### MTT Test

MTT test was performed on 5,000 NMuMG cells cultured in complete medium for 48h onto 96-well plates coated or not (PBS) with 333 pmol/cm^2^ (corresponding to 10 μg/cm^2^), 666 pmol/cm^2^ (20 μg/cm^2^) or 999 pmol/cm^2^ (30 μg/cm^2^) of purified recombinant human FBG-like domains. Cells were then incubated for 2h with 0.5 mg/mL MTT (3-(4,5-dimethylthiazol-2-yl)-2,5-diphenyltetrazolium bromide; Sigma-Aldrich) dissolved in culture medium, allowing NAD(P)H-dependent mitochondrial oxido-reductase enzymes to reduce MTT into formazan. Formazan was then solubilized from intracellular compartment using 10% (v/v) Triton X-100 in 0.1M HCl (Euromedex) and absorbance was measured by spectrophotometry at 570nm (Tecan M1000 PRO, infinite).

### Direct Fluorescence and Indirect Immunofluorescence Microscopy

100,000 NMuMG cells cultured in complete medium for 72h onto coverslips coated with purified recombinant human FBG-like domains (666 pmol/cm^2^) were fixed for 20min using 4% (w/v) paraformaldehyde, permeabilized for 10min using 0.5% (v/v) Triton X-100 in PBS and non-specific sites were saturated for 1h using 5% (v/v) SVF in PBS. All steps were performed at ambient temperature. Actin direct fluorescence analyses were performed using Tetramethylrhodamine isothiocyanate-labelled phalloidin (Sigma-Aldrich). Indirect immunofluorescence experiments were performed using mouse monoclonal anti-E-Cadherin (1/200; BD Transduction Laboratories™) and Alexa Fluor 488-conjugated goat anti-mouse IgG (1/1,000; Life Technologies) antibodies. DNA was stained with DAPI contained in the Vectashield mounting medium (Vector Laboratories). Observations were conducted using an ECLIPE Ti-E inverted microscope (Nikon) equipped with a DS-Fi2 color camera and the NIS element imaging software.

### Homology Modeling and Protein-Protein Docking

The four FBG-like domains of the human Tenascins, FBG-C, FBG-R, FBG-W and FBG-X, were modeled by homology using SWISS-MODEL web service. The PDB structure of the FGB-like domain of human TN-C (PDB code 6QNV chain A) was chosen as fold for the four models. The coverage for FBG-C starts at amino-acid #1979 to 2194 with 96% of identity, the FGB-R starts at amino-acid #1133 to 1358 with 60% of identity, FGB-W starts at amino-acid #1065 to 1280 with 52% of identity and for FBG-X starts at amino-acid #4025 to 4240 with 54% of identity. The high identity and the alignment quality of the FBG-like domain sequences with the fold were enough to create reliable models. All resulting models were checked, then charged and minimized using the MOE molecular modeling software (version 2019) with AMBER14:ETH force-field under pH 7. For latent TGF-β (pro-TGF-β1), PDB structure labeled 3RJR was used. This structure was checked, charged and minimized with the same protocol as for FGB-like domains.

Each FBG-like domain with pro-TGF-β1 were submitted to ClusPro webserver to perform protein-protein docking. No constraint in term of binding residue was set. 1,000 trials with rigid docking algorithms were selected and 30 cluster structures were constructed. Ten complexes optimized with charm force-field were output from the server. As complex is not mainly hydrophobic or hydrophilic, “balanced” score results were selected. For each FBG-like domain, the ten structures were checked, charged and minimized with MOE software using AMBER14:EHT force-field at pH 7.

### Molecular Dynamics and Trajectory Analysis

The twelve complexes were inserted in a TIP3P box, with a minimal distance of 12Å to the box limit, were prepared for molecular dynamic simulations. All simulations were performed using the molecular dynamics program AMBER18 (40) using the AMBER 14SB force-field. The simulation systems were kept under isothermal/isobaric (NPT) conditions except for the heating phase. Energy minimization was performed to obtain a low energy starting conformation for the subsequent MD simulation. The solvated complexes were minimized for a total of 5,000 cycles, using the steepest descent method for 2,500 cycles, followed by 2,500 cycles of a conjugate gradient. Then, a 1ns heating phase was performed from 0 to 300K at constant pressure and temperature. The equilibration/production was performed for 100ns. The time step of the simulations was 0.002ps.

The VMD software (41) was used to visualize trajectories generated during the simulation. Root Mean Square Deviation (RMSD) was used to determine structure stability. RMSDs were calculated for every simulation. Analysis of all RMSD reveals than the value of each dynamics is stabilized around 3Å and parameters of simulation reveal that simulations have reach equilibrium around 10 ns of simulation time. On the last 50ns of the trajectory 100 frames were sampled, one every 0.5ns.

### Affinity Binding Calculation and Alanine Scanning

For each simulation, the 100 sampled complexes without water were sent to Prodigy binding energy calculation. For each frame, the binding energy was computed and the mean energy was calculated. For each FBG, the three complexes conformation with the lowest binding energy were selected for additional analyzes. To investigate which residues of the structures were involved in the interaction, an alanine scanning simulation was performed. The last frame of MD for all three complexes was sent to MOE to do alanine scanning simulation. Each simulation will mutate to alanine all Tenascin amino-acid that may interact with latent TGF-β (pro-TGF-β1) or *vice versa*. Alanine scanning method was performed with a “rotamer explorer” algorithm with a conformation limit set to 50. The affinity was computed by GBVI/WSA algorithm between latent TGF-β and each FBG-like domain.

### Surface Plasmon Resonance (SPR) Analyses

The interaction between recombinant human mature TGF-β1 (dissolved in 10 mM citric acid; Peprotech) or human latent TGF-β1 (LTGF-β1, dissolved in PBS with 50% glycerol; ref. 299-LT-CF; BioTechne) and the four FBG-like domains or human Thrombospondin (TSP)-1 [purified from platelets as described (42)] was studied by surface plasmon resonance (SPR) using a Biacore T200 instrument. The four FBG domains and LTGF-β1 were immobilized on Series S CM5 sensorchips using amine coupling chemistry at pH 7.0 (in 10 mM HEPES) while FBG-X which was diluted in 10 mM sodium acetate pH 5.0. The control channels were prepared with the same activation/deactivation procedure except that the protein solution was replaced by buffer alone. The experiments were run in PBS buffer (Gibco) supplemented with 0.05% P-20 and sensorgrams were recorded at 25°C with a flow rate of 30 µl/min. Signals from control flow cells were automatically subtracted from signals in active flow cells. Regeneration of active and control channels was performed using 2 M NaCl. Kinetic data were analyzed using the Biacore T200 Evaluation software 3.2.

### Data and Statistical Analysis

The GraphPad Prism 8 software was used for graphical representations of data and statistical analyses. Each result shown is from an experiment representative of at least three independently repeated experiments. For MTT test, statistical analyses were performed using non-parametric ANOVA (Kruskal-Wallis test), and each condition was compared to the control condition (N-C) using Dunn’s multiple comparisons test. *p* values < 0.05 were considered as statistically significant (*p* values were indicated in the figure legends).

## Results

### Amino-Acid Sequence Conservation Between Tenascin FBG-Like Domains Suggests a Functional Redundancy in Latent TGF-β Activation

**Figure 1:**
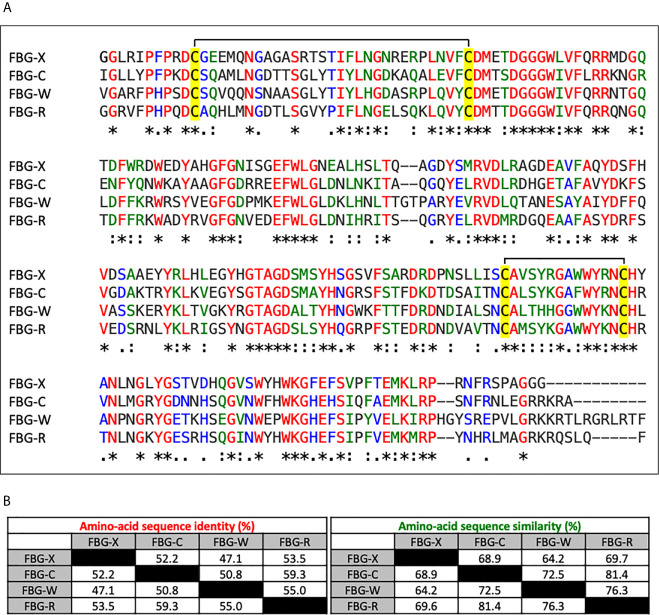
Sequence alignment and homology degree between the Tenascin family FBG-like domains. **(A)** Multiple sequence alignment analysis of the FBG-like domains of human TN-X, -C, -W and -R (FBG-X, -C, -W, -R) performed with CLUSTALW software. Red (*) labelled residues are identical between the four FBG-like domains, green labelled (:) residues are highly conserved and blue labelled (.) residues are poorly conserved. The four conserved cysteine residues are highlighted in yellow. **(B)** Percentages of identity and similarity amongst the amino-acid sequences of the FBG-like domains of the human Tenascins performed with paired comparisons using CLUSTALW software.

To evaluate sequence conservation between the four human Tenascin FBG-like domains (hereafter called FBG-C, FBG-R, FBG-W and FBG-X), their amino-acid sequences were aligned using the CLUSTALW software (**Figure 1A**). Sequence comparison showed that the FBG-like domains of the four Tenascins share a high degree of identity (35 %) and similarity (55 %) when compared all together (**Figure 1A**). This high degree of sequence homology may also imply a structural similarity, because the cysteine residues responsible for the formation of two disulfide bounds are strictly conserved between the four domains (**Figure 1A**, highlighted in yellow). In order to perform a deeper analysis, we also compared the sequences of FBG-like domains in pairs (**Figure 1B**). When compared two by two, the degree of similarity is strongly increased, showing that FBG-like domains share more than 50% of sequence identity and at least 65% of similarity, except for FBG-X and FBG-W, which appeared to be the most divergent ones. Consequently, FBG-C seems to be closer to FBG-R and FBG-W, and FBG-X seems to be the most distant domain of the Tenascin family (still with >47% identity with its counterparts), as previously suggested by Chiquet-Ehrismann (43). Nevertheless, this high degree of homology between FBG-like domains strengthens the idea that latent TGF-β activation might be a common property to all members of the Tenascin family.

### Recombinant Full-Length Tenascins and Their Respective FBG-Like Domains Are Produced and Purified in Association With the Small Latent Complex

**Figure 2:**
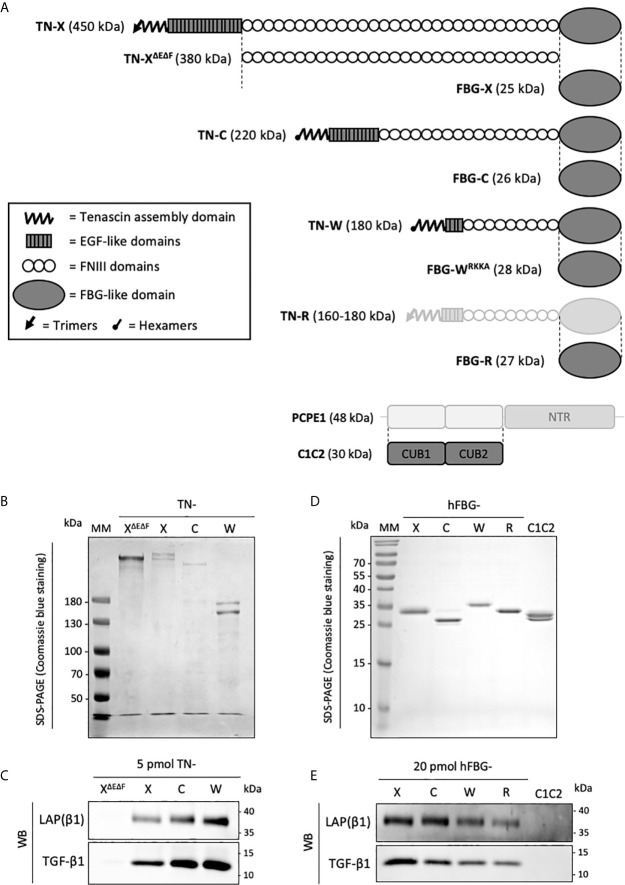
Recombinant full-length Tenascins and their respective FBG-like domains co-purify with latent TGF-β1. **(A)** Schematic representation of the recombinant proteins used in this study (dark grey). Structural modules of Tenascins are depicted in the inset. **(B, D)** SDS-PAGE analysis of the purified recombinant proteins and stained with Coomassie blue. MM, molecular mass markers. **(B)** Purified full-length TN-X, TN-C, TN-W and the TN-X^ΔEΔF^ fragment (1 μg each) were loaded on 6% acrylamide gels under reducing conditions. **(D)** Purified recombinant FBG-like domains of the four human Tenascins and the recombinant CUB1CUB2 (C1C2) domain (2 μg) were resolved on 15% acrylamide gels under reducing conditions. **(C, E)** Western blot analysis indicating the level of human mature TGF-β1 and LAP(β1) pro-domain associated with equimolar quantity of purified recombinant full-length TN or TN-X^ΔEΔF^ fragment (5 pmol each) **(C)** and recombinant Tenascin FBG-like domains or CUB1CUB2 (C1C2) protein (20 pmol) **(E)**.

We first wanted to determine whether human TN-C and TN-W could be associated with latent TGF-β, as previously observed for TN-X (28). To do so, recombinant full-length TN-C (220 kDa), TN-W (180 kDa) and TN-X (450 kDa) were produced in mammalian cells and purified from conditioned media using specific chromatographic steps as detailed in the “Materials and Methods” section (**Figures 2A, B**). Full-length TN-R was not included in this study because, to our knowledge, no recombinant human TN-R has been cloned and characterized yet. As negative control, we also included a recombinant TN-X fragment called TN-X^ΔEΔF^ (380 kDa), which does not contain EGF-like repeats and FBG-like domain (**Figure 2A**) (3). This truncated TN-X fragment is used to indicate the basal level of contaminating TGF-β1 in our purified recombinant proteins (28). We then decided to analyze the presence of SLC components (*i.e.* TGF-β and LAP(β) entities) in purified protein fractions. Immunoblotting performed on equimolar quantity of purified TN-X, TN-C and TN-W confirmed the presence of both TGF-β1 moiety and LAP(β1) pro-domain in all recombinant full-length protein preparations (**Figure 2C**), showing that these three glycoproteins co-purify with the endogenously synthesized latent TGF-β1. As previously described, TN-X^ΔEΔF^ purification fraction does not contain any component of the SLC (**Figure 2C**), thus confirming that latent TGF-β1 is not associated with TN-X FNIII repeats.

To confirm the implication of the C-terminal globe of Tenascin family members in this association, FBG-like domains of human TN-X, -C, -W, and -R were cloned, produced in mammalian cells, and purified from conditioned medium using nickel-chelating chromatography (**Figures 2A, D**). As negative control, we used a purification fraction of CUB1CUB2 (C1C2), a recombinant histidine-tagged fragment derived from the Procollagen C-proteinase enhancer-1 (PCPE-1) extracellular protein. The recombinant C1C2 fragment was produced using similar conditions as for the FBG-like domains (mammalian host cells and purification method) (38) and has a comparable molecular mass (≈30 kDa). After protein purity assessment by Coomassie Blue staining on SDS-PAGE (**Figure 2D**), we performed immunoblotting on equimolar quantity of purified FBG-like domains or C1C2 protein to analyze the presence of SLC components. As shown in **Figure 2E**, both TGF-β1 and LAP(β1) entities were detected in each purified FBG-like domain fraction, and were absent from C1C2 fraction, confirming the specificity of the molecular association between latent TGF-β1 and Tenascin FBG-like domains. Altogether, these results indicated that the Tenascin family members might be specifically associated with latent TGF-β1 through their FBG-like domains.

During the course of the experiments, we identified a cleavage site located within the C-terminal sequence of the human FBG-W domain that is responsible for the removal of the histidine tag during the biochemical purification of the recombinant protein (**Figure S1**). Indeed, this domain harbors a cationic tail with the following sequence Arg^1,217^-Lys-Lys-Arg^1,220^ (RKKR), which corresponds to the consensus sequence (Arg-Xaa-(Lys/Arg)-Arg-Xaa) for the Furin-like family of pro-protein convertases. Using site-directed mutagenesis, we abolished the C-terminal cleavage site by substituting the Arg^1,220^ amino-acid by an alanine residue. Compared to the FBG-W^RKKR^ domain, the molecular mass of the FBG-W^RKKA^ protein is higher and the histidine-tag is henceforth detectable (**Figure S1**). As this substitution does not prevent the co-purification of the modified FBG-W domain with the latent TGF-β (**Figure 2E**), this recombinant version was used throughout the study. This result also suggests that the molecular association between the FBG-like domains and the LAP–TGF-β complex does not involve the very C-terminal extremity of the FBG knob.

### The Recombinant FBG-Like Domains of the Four Tenascins Physically Interact With TGF-β

**Figure 3:**
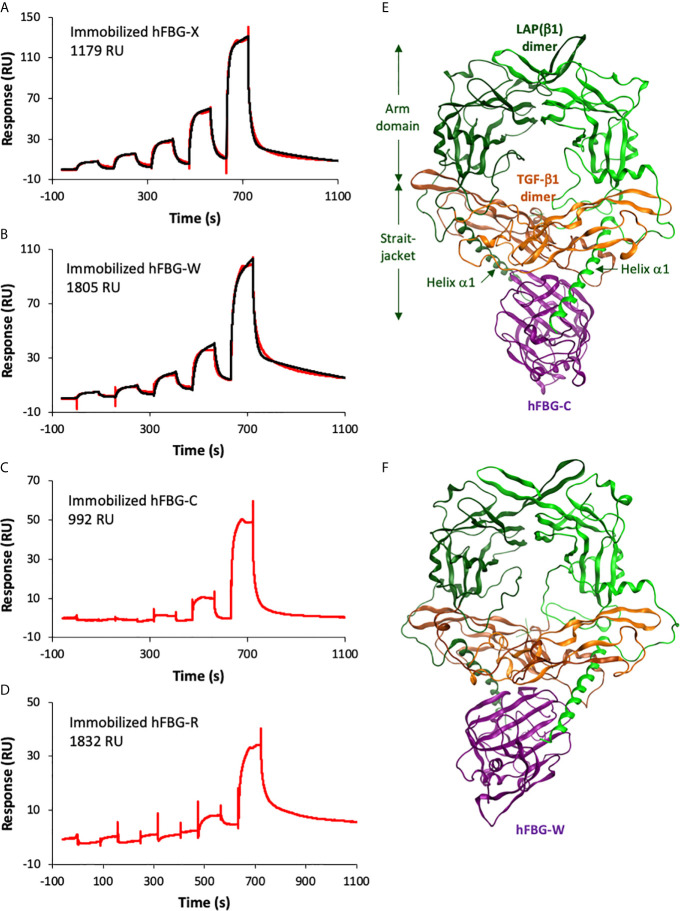
The recombinant FBG-like domains of Tenascins interact with TGF-β1. **(A–D)** Interactions of TGF-β1 with FBG-like domains analyzed by SPR. Increasing concentrations of TGF-β1 were injected in single-cycle mode over immobilized FBG-X **(A)**, FBG-W **(B)**, FBG-C **(C)** and FBG-R **(D)**. The experimental curves are shown in red and the best fits with the heterogeneous ligand model in black. The concentrations of TGF-β1 were as follows: **(A)** 15.6 – 31.2 – 62.5 – 125 – 250 nM; **(B)** 7.8 – 15.6 – 31.25 – 62.5 – 125 nM; **(C)** 20.2 – 40.5 – 81 – 162 – 324 nM; **(D)** 9.4 – 18.8 – 37.5 – 75 – 150 nM. **(E, F)** Homology models showing the predicted docking between the FBG-like domain of TN-C **(E)** or TN-W **(F)** and the pro-TGF-β1. The dimeric LAP(β1) pro-domain is shown in green, the TGF-β1 dimer in orange and the FBG-like domains in purple. ‘Straitjacket’ and arm domains of the LAP(β1) dimer are indicated.

To confirm a physical interaction between the four FBG-like domains and the small latent (LAP–TGF-β) complex, surface plasmon resonance analyses (SPR) were performed using commercially available recombinant human latent TGF-β1 (LTGF-β1). Although we previously demonstrated that LAP(β1) co-immunoprecipitated with FBG-X (28), we failed to detect an interaction between the four FBG-like domains of Tenascins and recombinant LTGF-β1 by SPR (**Figure S2**). As we did not succeed either to show the binding of LTGF-β1 with purified human TSP-1 (**Figure S2B**), a well-established partner and activator of latent TGF-β (44), it remains questionable whether the purified recombinant LTGF-β1 was in a favorable conformation to enable such an interaction using this biochemical approach. In contrast, we demonstrated a direct interaction between mature TGF-β1 and the four FBG-like domains (**Figures 3A–D**). The association and dissociation curves obtained with different concentrations of TGF-β1 were best fitted with the heterogenous ligand model to determine the apparent equilibrium dissociation constants (K_D_) between the different entities (**Table 3**). Bioactive TGF-β1 binds with higher affinity to FBG-X (K_D1 =_ 342 nM and K_D2 =_ 407 nM) than to FBG-W (K_D1 =_ 1.9 µM and K_D2 =_ 6.5 µM). Curves derived from the interaction between TGF-β1 and immobilized FBG-C and FBG-R could not be fitted due to weak signals at the lower TGF-β1 concentrations and aggregation occurring when higher concentrations were injected.

**Table 3:** Kinetic and dissociation constants derived from SPR analysis.

	k_a1_ (M^-1^s^-1^)	k_d1_ (s^-1^)	K_D1_ (µM)	k_a2_ (M^-1^s^-1^)	k_d2_ (s^-1^)	K_D2_ (µM)	Chi^2^ (RU^2^)
hFBG-X	8085	0.00276	**0.342**	2.074 10^5^	0.0845	**0.407**	5.27
hFBG-C	*NA*	*NA*	*NA*	*NA*	*NA*	*NA*	*NA*
hFBG-W	1036	0.00196	**1.89**	1.430 10^4^	0.0926	**6.48**	4.21
hFBG-R	*NA*	*NA*	*NA*	*NA*	*NA*	*NA*	*NA*

See legend of **Figure 3** for experimental conditions. Best fits for the interactions of TGF-β1 with FBG-X and FBG-W were obtained with the heterogeneous ligand model. NA, not applicable.

### Molecular Docking of the Small Latent Complex With the FBG-Like Domains of the Tenascins

The latter result prompted us to model the association of pro-TGF-β1 (31) with the four human Tenascin FBG-like domains using modeling and molecular dynamic approaches. Thanks to the high sequence identity observed between the four FBG-like domains, it was possible to generate reliable models of the FBG-R, -W and -R from the known X-ray structure of FBG-C (PDB code 6QNV). Indeed, homology models predict that each FBG-like domain exhibits similar subdomain organization and folding. Then, protein-protein docking simulations for the complexes involving FBG-like domains and pro-TGF-β1 were performed with ClusPro. For each FBG-like domain, three poses with the best score affinity were selected (**Figures 3E, F** and **S3A**). Binding energies between the four domains revealed some differences, FBG-X displaying the highest (14.2, 14.8, and 13.2 kcal.mol^-1^), FBG-W (13.2, 11.9 and 13.1) and FBG-C (12.8, 11.1, and 12.4 kcal.mol^-1^) intermediate, and FBG-R (10.7, 11.2, and 11.7 kcal.mol^-1^) the lowest values. These energy scores suggested that FBG-X should bind with a higher affinity to pro-TGF-β1 than FBG-W and FGB-C, themselves having a better affinity than FBG-R. The twelve selected complexes were submitted to an alanine scanning simulation and resulting binding energies were further calculated. For each protein domain, residues were considered to play a critical role in the interaction if the ΔΔG affinity between the wild-type FBG and the corresponding virtual mutant was higher than 2 kcal.mol^-1^ and if they came out in the three simulations performed with a specific FBG-like domain. These simulations predicted a limited number of key residues (four to six residues per domain) involved in the interaction with the FBG-like domains. These are mostly located in the loop 9 of the four FBG-like domains, a region located at the C-terminal part of the domains, but upstream of the cationic tail of FBG-W, FBG-C and FBG-R (**Figure S3B**). These residues are predicted to play a critical role in the interaction with pro-TGF-β1 mostly through hydrogen bonds and steric modification of the interface. In addition, the pro-TGF-β1 is suggested to interact with the FBG-like domains not only through the LAP pro-domain (helix α1 from the straightjacket domain) but also with the N-terminal region of the mature TGF-β1 moiety (**Figures 3E, F**, **S3A**, **S4**). Pro-TGF-β1 was also submitted to an alanine scanning simulation and critical residues predicted to be involved in the interaction with each FBG-like domain were also determined (**Figure S4**).

### FBG-Like Domains of the Tenascin Family Members Activate the Canonical TGF-β/Smad Intracellular Signaling Pathway

**Figure 4:**
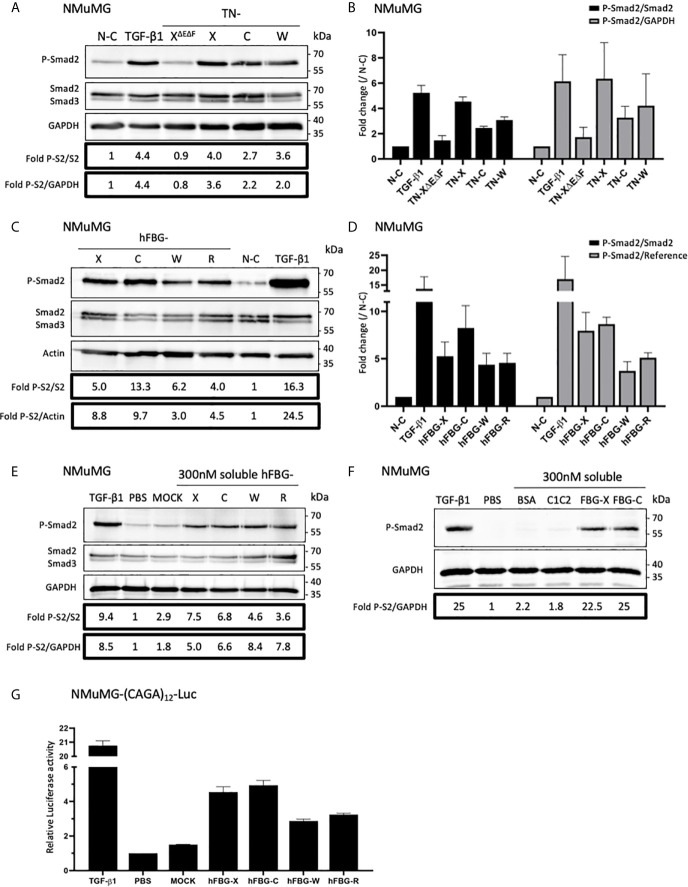
Full-length Tenascins and their respective FBG-like domains stimulate TGF-β/Smad intracellular signaling pathway in epithelial cells. **(A)** Western Blot analysis showing phosphorylated Smad2 (P-Smad2), total Smad2/3 and GAPDH levels in NMuMG cells cultured for 3h onto control non-coated (N-C) dishes or dishes coated with equimolar quantity (22,2 pmol/cm^2^) of full-length Tenascins (TN) or central TN-X fragment (TN-X^ΔEΔF^), or stimulated with soluble active TGF-β1 (2 ng/mL). **(B)** Fold changes of P-Smad2 to total Smad2 levels or to GAPDH levels. Graph shows means ± SD of n = 3 independent experiments. **(C)** Western Blot analysis showing P-Smad2, total Smad2/3 and Actin levels in NMuMG cultured for 3h onto equimolar quantity of FBG-like domains (333 pmol/cm^2^) or stimulated with soluble active TGF-β1 (2 ng/mL). **(D)** Fold changes of P-Smad2 to total Smad2 levels or to reference protein levels. Graph shows means ± SD of n = 3 independent experiments. **(E)** Western-blot analysis showing P-Smad2, Smad2/3 and GAPDH levels in NMuMG cells stimulated for 1h in the presence of active TGF-β1 (2 ng/mL), stimulated or not (PBS) with soluble FBG-like domains (300 nM) or MOCK-CM. “MOCK-CM” referred to the conditioned media of MOCK-transfected HEK293 EBNA cells, submitted to Nickel-affinity chromatography and eluted from the column as for the FBG-like domains. **(F)** Immunoblotting analysis showing P-Smad2 and GAPDH levels in NMuMG cells stimulated for 1h in the presence of active TGF-β1 (2 ng/mL), stimulated or not (PBS) with soluble CUB1CUB2 protein fragment (C1C2), bovine serum Albumin (BSA) or FBG-like domains (300 nM each). **(G)** Relative Luciferase activity of NMuMG cells transiently transfected with the Smad-responsive (CAGA)_12_-Luc reporter construct and treated for 16h with soluble FBG-like domains (600 nM), MOCK-CM or TGF-β1 (2 ng/mL). Graph shows one representative result from 3 independent experiments.

Because of their association with latent TGF-β1, we investigated the ability of full-length Tenascins and FBG-like domains to stimulate the canonical TGF-β/Smad intracellular signaling pathway. In the first set of experiments, we analyzed the phosphorylation of Smad2 in Normal Murine Mammary Gland (NMuMG) epithelial cells seeded for 3h onto non-coated (N-C) dishes, or dishes coated with equimolar quantity of recombinant proteins (22,2 pmol/cm^2^ for full-length Tenascins or 333 pmol/cm^2^ for FBG-like domains). Because cells were not able to adhere properly onto full-length TN-C due to its anti-adhesive property (45), detached NMuMG cells were harvested and pelleted from culture medium prior to protein extraction. While NMuMG cells cultured onto N-C dishes or dishes coated with TN-X^ΔEΔF^ displayed basal levels of phosphorylated Smad2, cells cultured onto full-length TN-X, TN-C and TN-W exhibited a marked phosphorylation of Smad2 (**Figure 4A**). Smad2 phosphorylation is comparable to that obtained with TGF-β1 stimulation for cells cultured onto TN-X and slightly less intense (but still strong) for cells cultured onto immobilized TN-C and TN-W (**Figures 4A, B**). Level of Smad2 phosphorylation was also increased when NMuMG cells were seeded onto equimolar quantity of immobilized FBG-like domain of the four Tenascins (**Figures 4C, D**).

Because Tenascins are known to modulate cell adhesion, we next decided to analyze Smad activity in NMuMG cells stimulated with soluble, instead of immobilized, recombinant proteins. Results obtained with equimolar concentration of soluble FBG-like domains were similar to those obtained using coated proteins, resulting in Smad2 phosphorylation in NMuMG cells (**Figure 4E**). As a negative control, we used the conditioned medium obtained from mock-transfected cells that was submitted to a nickel-chelating column (MOCK), as done for the FBG-like domains. NMuMG stimulation using this mock fraction induced a near to basal Smad2 phosphorylation (**Figure 4E**). Similarly, the CUB1CUB2 protein fragment failed to induce an activation of the Smad signaling pathway in NMuMG cells (**Figure 4F**), thus confirming that Smad2 phosphorylation can be attributed to the Tenascin-derived proteins, but not to an irrelevant protein domain.

As a readout of Smad2 transcriptional activity, we performed Luciferase analyses in NMuMG cells. Cells were transiently transfected with the (CAGA)_12_-Luc synthetic reporter construct containing twelve repeats of the Smad-binding element (39) and were stimulated with soluble recombinant FBG-like domains. Recombinant human TGF-β1 and MOCK or vehicle (PBS) were respectively used as positive and negative controls. Stimulation with soluble FBG-like domains induced a marked increase of luciferase activity in transiently transfected NMuMG cells, in comparison with MOCK or PBS treatment (**Figure 4G**). Altogether, these results indicated that the FBG-like domains of the four Tenascins induced a canonical TGF-β/Smad intracellular pathway in epithelial cells, resulting in a functional Smad transcriptional activity.

### Tenascins Are Able to Activate Latent TGF-β Thanks to Their FBG-Like Domains

**Figure 5:**
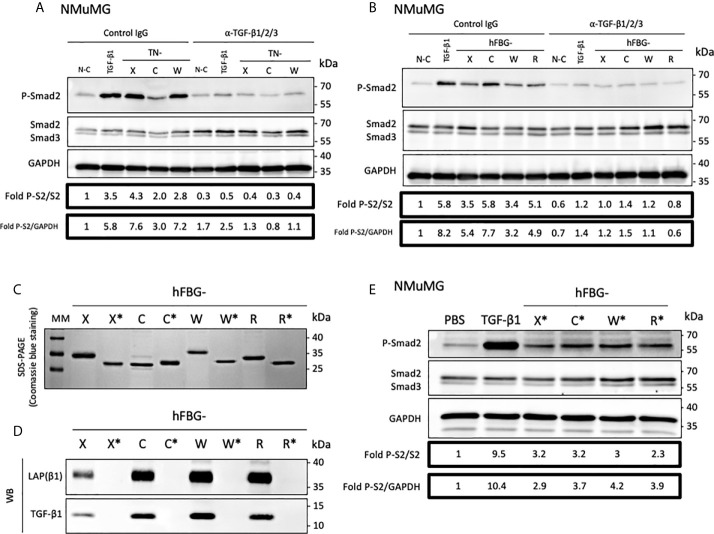
The FBG-like domain of the Tenascin family members activates the latent TGF-β in epithelial cells. **(A)** Western-blot analysis showing P-Smad2, total Smad2/3 and GAPDH levels in NMuMG cultured for 3h onto Non-Coated (N-C) dishes, dishes coated with 22,2 pmol/cm^2^ full-length Tenascins (TN) or stimulated with 2 ng/mL TGF-β1, in presence of anti-pan-TGF-β antibody or isotype-matched control IgG (10 μg/mL). **(B)** Western Blot analysis showing P-Smad2, total Smad2/3 and GAPDH levels in NMuMG cells cultured for 3h in presence of 333 pmol/cm^2^ of FBG-like domains as described in **(A)**. **(C)** SDS-PAGE analysis of purified recombinant FBG-like domains produced in mammalian cells (FBG) and *E. coli* (FBG*) resolved on 15% acrylamide gels under reducing conditions (2 µg each). MM, molecular mass markers. **(D)** Western-Blot analysis of the levels of mature TGF-β1 and LAP(β1) pro-domain associated with 20 pmol of FBG-like domains produced in mammalian cells (FBG) and *E coli* (FBG*). **(E)** Western-blot analysis showing P-Smad2, Smad2/3 and GAPDH levels in NMuMG cells stimulated for 1h with active TGF-β1 (2 ng/mL), soluble purified FBG-like domains produced in *E Coli* (720 nM) or vehicle (PBS).

Then, we planned to determine whether TN-X ability to activate latent TGF-β through its C-terminal knob was shared by TN-C and TN-W. We first analyzed the impact of full-length Tenascins on the canonical TGF-β signaling pathway in the presence of an anti-TGF-β1/2/3 neutralizing antibody directed against mature TGF-β entities. In NMuMG cells, this antibody significantly inhibited Smad2 phosphorylation induced by either soluble mature TGF-β1 or immobilized full-length Tenascins, as compared with control IgG (**Figure 5A**). Similarly, the neutralizing antibody abolished Smad2 phosphorylation provoked by coated FBG-like domains (**Figure 5B**). These results suggest that FBG-like domains of the four Tenascins are able to induce Smad signaling by increasing mature TGF-β bioavailability.

The FBG-like domains produced in mammalian cells are associated with latent TGF-β1 (**Figure 2E**). To get rid of this source of endogenous TGF-β and to determine whether FBG-like domains are able to activate exogenous latent TGF-β (*i.e.* secreted by the cells or from the serum), we produced these recombinant domains in a prokaryotic system. As shown by SDS-PAGE coupled with Coomassie blue staining (**Figure 5C**), the FBG-like domains produced in bacteria (noted FBG*) displayed lower molecular masses than those produced in mammalian cells, most likely due to the absence of post-translational modifications in the proteins produced in prokaryotic systems. Although purified recombinant FBG-X*, FBG-C*, FBG-W* and FBG-R* did not contain any TGF-β1 and LAP(β1) (**Figure 5D**), they retained their ability to induce Smad2 phosphorylation when presented to NMuMG cells in a soluble form (**Figure 5E**). To ensure that Smad2 phosphorylation was solely attributed to FBG-like domains, but not to bacterial contaminants such as lipopolysaccharides (LPS) and LPS-associated molecules, we confirmed that increasing doses of LPS (1-100 ng/mL) did not induce Smad2 phosphorylation in NMuMG cells (**Figure S5**). These experiments definitively showed that the FBG-like domains produced in bacteria and devoid of TGF-β kept their ability to activate the pool of latent TGF-β that is either secreted by cells or available from the serum, resulting in the induction of a TGF-β/Smad intracellular pathway.

### FBG-Like Domain of Tenascins Trigger EMT and Cytostasis in Epithelial Cells

**Figure 6:**
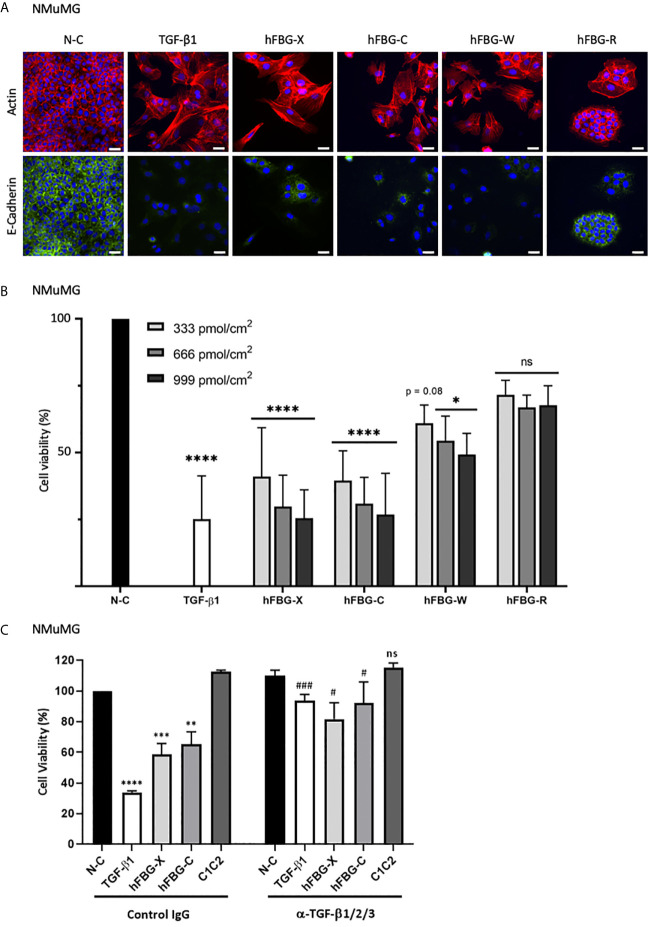
The FBG-like domains of Tenascins trigger EMT and cytostasis in epithelial cells. **(A)** F-actin direct fluorescence (red) and E-Cadherin indirect immunofluorescence (green) performed on NMuMG cells cultured for 72h onto N-C dishes, dishes coated with 666 pmol/cm^2^ FBG-like domains or stimulated with soluble TGF-β1 (5 ng/mL). Cell nuclei were counterstained with DAPI (blue). Bars, 15 µm. **(B)** Percentage of cell viability in NMuMG cells cultured for 48h onto N-C dishes, dishes coated with 333, 666 or 999 pmol/cm^2^ FBG-like domains, or stimulated with 5 ng/mL TGF-β1. Error bars are means ± SD from 3 independent experiments. *p < 0.05 compared to N-C condition. ****p < 0.0001 compared to N-C condition. ns, not significant. **(C)** Percentage of cell viability in NMuMG cells cultured for 48h onto N-C dishes, dishes coated with 666 pmol/cm^2^ FBG-like domains (FBG-X and FBG-C) or CUB1CUB2 protein fragment (C1C2), or stimulated with 5 ng/mL TGF-β1, in the presence of anti-pan-TGF-β antibody or isotype-matched control IgG (5 μg/mL). Error bars are means ± SD from 3 independent experiments. **, *** and **** respectively correspond to *p* < 0.01, *p* < 0.001 and *p* < 0.0001 compared to N-C condition. ^#^ and ^###^ respectively correspond to *p* < 0.05 and *p* < 0.001 *versus* their control IgG-treated counterpart. ns, not significant.

Cytostasis and epithelial-to-mesenchymal transition (EMT) are two cellular programs promoted by bioactive TGF-β (46–48). Firstly, to determine whether the activation of latent TGF-β mediated by the four FBG-like domains was sufficient to regulate epithelial cell plasticity, we compared actin and E-Cadherin localizations in NMuMG cells cultured onto N-C dishes (control) or dishes coated with the FBG-like domains. In control condition, NMuMG cells displayed a cuboidal shape, with cortical actin cytoskeleton distribution and E-Cadherin localization at the cell-cell junctions, two typical features of an epithelial cell morphology (**Figure 6A**). As expected, NMuMG cells stimulated with soluble mature TGF-β1 or cultured onto immobilized FBG-X underwent EMT, characterized by a cellular scattering, a reorganization of actin cytoskeleton into stress fibers and a loss of E-Cadherin signal. Interestingly, epithelial cells cultured onto dishes coated with recombinant FBG-C, FBG-W or FBG-R also displayed EMT features, but at different degrees. Even if a pool of actin remained cortically located, a fraction of F-actin reorganized to form stress fibers, whereas E-Cadherin disappeared from the cell-cell junctions. This phenotypical switch was partially observed in NMuMG cultured onto coated FBG-R, in which some cellular islets still exhibited epithelial features, while others underwent partial EMT (**Figure 6A**). Concomitantly, quantitative gene expression analyses revealed a decrease of epithelial cell marker (*E-cadherin*) and a gain of mesenchymal cell markers (*Vimentin* and *Fibronectin-1*), as well as of EMT inducers (*Hmga2*, *Snail1*, and *Zeb1*), when cells were cultured onto immobilized FBG-like domains. Once again, transcriptional responses varied among the FBG domain, the strongest being observed with the FBG-X and the lowest with FBG-R (**Figure S6A**). The EMT transcriptional program induced by the FBG-like domain is abolished in the presence of a neutralizing antibody, thus confirming that this cell response is dependent on TGF-β activation (**Figure S6B**).

A concomitant observation with EMT was the marked decrease in cell number, as judged by nuclei staining (**Figure 6A**), when NMuMG cells were cultured onto immobilized recombinant FBG-like domains or stimulated with active TGF-β1. We then assessed whether the activation of latent TGF-β mediated by the four FBG-like domains was able to induce a cytostatic program in epithelial cells. As a readout of cell number, we performed a MTT assay in NMuMG cells stimulated with recombinant active TGF-β1 or cultured onto dishes coated with increasing quantities of FBG-like domains (*i.e.* 333, 666 and 999 pmol/cm^2^) (**Figure 6B**). While FBG-X and FBG-C induced a strong reduction of living cell number comparable to TGF-β1 for the highest quantity (999 pmol/cm^2^), FBG-W and FBG-R led to a lower but still significant reduction of cell number. The decrease in numbers of living cells was proportional to the quantity of proteins used in this experiment (**Figure 6B**). The reduction of cell number in the presence of the FBG-like domains was not the consequence of a cytotoxic effect due to the cell exposure to an exogenous protein domain, as the irrelevant CUB1CUB2 protein fragment did not modify cell viability (**Figure 6C**). Finally, the decrease in living cell number could be attributed to the cytostatic effect of bioactive TGF-β, as the neutralizing anti-mature TGF-β1/2/3 antibody significantly reverted the effects mediated by the FBG-like domains (**Figure 6C**).

Altogether, these results indicated that the canonical Smad intracellular pathway induced by the FBG-like domains of the four Tenascins resulted in the activation of TGF-β cell programs in mammary epithelial cells, such as EMT and growth arrest.

## Discussion

Because of its central role in tissue homeostasis and integrity, extracellular activation of latent TGF-β is a crucial mechanism that needs to be tightly regulated (49, 50). By interacting with several ECM components, the small (SLC) and the large (LLC) latent complexes are trapped within the matrix compartment, creating a pool of latent cytokine that can be activated upon requirement. Even if a growing number of ECM proteins are referenced as TGF-β partners (29), only a few have the ability to promote its activation. To our knowledge, Thrombospondin-1 (TSP-1), Connective Tissue Growth Factor (CTGF, also known as CCN2) and TN-X have been so far the only three ECM macromolecules described as latent TGF-β activators, both *in vitro* (28, 51, 52) and *in vivo* (52, 53).

In this study, we confirmed the TN-X ability to activate latent TGF-β and identified human TN-C, TN-W and possibly TN-R, as new activators of the latent cytokine *in vitro*, thanks to their highly conserved C-terminal FBG-like domain. Indeed, we found that the FBG-like domains of TN-C, TN-W and TN-R are associated with TGF-β1 SLC *in vitro* and that this molecular association promotes the presentation of the mature cytokine to cells. This conclusion first relies on the fact that FBG-like domains of the four Tenascins co-purified with both entities of the SLC, *i.e.* TGF-β1 and its LAP pro-domain (**Figure 2**). Second, the globular domains of TN-C, TN-W and TN-R physically interact with TGF-β1 *in vitro* (**Figure 3**). Third, the FBG-like domains are able to unravel the mature TGF-β1, as they induce a Smad intracellular signaling pathway activation when presented to cells (either in an immobilized or a soluble form, **Figure 4**). This activation leads to the induction of TGF-β-responsive programs, such as EMT and cytostasis in epithelial cells, two processes that are abolished by the presence of a neutralizing anti-mature TGF-β1/2/3 antibody (**Figure 6** and **S6**). Finally, FBG-like domains produced in bacteria and free from endogenous TGF-β are able to activate the latent cytokine from an exogenous source, either present in the serum or secreted by cells (**Figure 5**).

Although the concomitant binding of the LAP pro-domain to the TGF-β–FGB protein complex remains to be confirmed experimentally for TN-C, TN-W and TN-R, molecular modeling and dynamic analyses predicted the interaction of the small latent (LAP–TGF-β) complex with the FBG-like domain of the four Tenascins (**Figures 3E, F** and **S3**). More specifically, alanine scanning analyses predict that the dimeric latent TGF-β ‘sits’ on the C-terminal portion of the FBG globes, through interactions with both the LAP pro-domain and the mature TGF-β moiety (**Figures 3E, F** and **S3**), thus giving insight into the biochemical association observed between these two entities and the FBG-like domains. The dimeric LAP(β1) pro-domain is predicted to bind to the FBG-like domain, through several amino acids from the first α-helix that are located immediately upstream of the latency lasso, which maintains mature TGF-β1 in a latent form through non-covalent interactions (31). In addition, the dimeric mature TGF-β1 is suggested to bind to the FBG-like globes through three stretches of sequences located upstream or surrounding the interchain disulfide bond (Cys^355^). In turn, five to six key amino-acid residues mostly located to the loop9 of the FBG-like domains are predicted to bind with pro-TGF-β1 mostly through hydrogen bonds and steric modifications of the interface. The involvement of these candidate amino acids in the interaction needs further validation not only *in vitro* using mutated recombinant FBG-like domains, but also *in vivo* with the analysis of inherited variants occurring in the globular domains. Indeed, Morissette and collaborators identified a novel *TNXB* c.12174C>G mutation that reduces the binding affinity of latent TGF-β to the native TN-X (54). This mutation, resulting in the p.C4058W amino-acid substitution within the FBG-like knob of TN-X, is not located in the loop9, but resides in its N-terminal portion. This cysteine residue, which is involved in the formation of a first disulfide bond within the FBG-like domain, is conserved not only in TN-X proteins from different species, but also in other members of the human Tenascin family (cysteine in position 11, **Figure 1A**). The first disulfide bridge is predicted to maintain the correct orientation of the FBG-like domain downstream of the FNIII modules. The absence of this disulfide bond in TN-X^C4058W^ might result in an inappropriate folding of the FBG-like domain over the FNIII modules, thus preventing an optimal presentation of this domain towards its molecular partner. Further molecular dynamic analyses are needed to confirm this hypothesis.

SPR analyses confirmed the direct interaction between mature TGF-β1 and the four FBG-like domains (**Figures 3A–D**). For both FBG-X and FBG-W, kinetics sensorgrams were best fitted with the heterogenous ligand model, indicating that TGF-β1 interaction may result from two conformations of the globular domain on the sensor chip or may occur with at least two binding sites on the FBG-like domains. Notably, the two binding sites display similar dissociation constants for one FBG but result from kinetic constants that differ by one log. This model is also compatible with a putative conformational change triggered by the FBG-like domain on the latent cytokine, as explained hereafter. The kinetics data indicate that TGF-β1 binds to FBG-X with a higher affinity than to FBG-W. This experimental observation correlates with the calculated binding energies for the four domains. Indeed, *in silico* analyses predict that FBG-X displays the highest affinity for pro-TGF-β1, then followed by FBG-W and FBG-C, with FBG-R having the lowest value. These relative affinities, obtained in biochemically defined environment (SPR) or from *in silico* analyses (molecular modelling), are rather consistent with the intensity of the cellular responses obtained *in vitro* for each FBG-like domain. Indeed, FBG-X mostly exhibited the strongest TGF-β-dependent response, compared to FBG-W and FBG-C, while FBG-R was the recombinant domain displaying the weaker effect. In line with this, the binding parameters of FBG-C and FBG-R could not be determined because of the weak signals obtained with the lowest TGF-β1 concentrations, indicating a weaker affinity of these two FBG-like domains for the mature cytokine. Moreover, kinetics analyses were also limited in the high concentration range by the fact that mature TGF-β1 tended to form aggregates at higher concentrations.

These observations raise further questions regarding the exact role(s) of full-length Tenascins in the regulation of TGF-β bioavailability in a complex tissue environment. Are full-length molecules only involved in the storage of the latent cytokine within the ECM or do they also participate in the activation of latent TGF-β *in vivo*? In our *in vitro* assays we noticed that FBG-like domains appeared to be less effective at inducing Smad2 phosphorylation than full-length Tenascin proteins (**Figure 4**). Although it is conceivable that a hexameric molecule (TN-C or TN-W) might offer a 2-fold higher probability to bind to and to activate latent TGF-β compared to a trimeric protein (TN-X or TN-R) *in vivo*, it is unlikely the case in our *in vitro* assays. Indeed, cells were seeded onto equimolar quantity of molecules (with regard to the molecular mass of each monomeric chain), *i.e.* onto similar number of FBG-like domains, whatever the length (intact or solely the C-terminal portion of Tenascin) or the assembly (oligomeric or monomeric) of the immobilized protein. However, the oligomeric nature of the proteins might help in the activation process, by offering multiple and cooperative binding sites to latent TGF-β. Although less compatible with the molecular modeling analyses, it is possible that two FBG-like domains from independent chains within an oligomer might cooperate in binding to and activating latent TGF-β (or presenting activated TGF-β to cell-surface receptors). While analyzing the interaction between FBG-like domains and mature TGF-β1 using SPR, the bivalent analyte model also gave good fits of the experimental curves (**Table S1**). These observations suggest that a single dimeric mature TGF-β molecule might bind to two FBG-like domains simultaneously. Alternatively, it is also possible to propose a cooperation between the FBG-SLC complex with another TGF-β-binding site within the full-length molecule in the process of latent TGF-β activation. For instance, the fifth FNIII domain of TNC has also been shown to interact with TGF-β1 (55). One can speculate that in an hexameric TN-C protein, one FBG-like domain from one chain may cooperate with a fifth FNIII domain (from the same or an independent chain) in the activation of the latent cytokine. However, this hypothesis in unlikely true for TN-X as the central region of this glycoprotein (the TN-X^ΔEΔF^ fragment only composed of FNIII domains) does not interact neither with the LAP(β1) pro-domain nor with the mature TGF-β1 (this study and (28)). Finally, we cannot exclude in our experiments that the small globular FBG-like proteins may be less efficiently adsorbed to plastic culture dishes than to the full-length Tenascin molecules in regards with their intrinsic physicochemical properties and/or structural parameters.

Extracellular activation of latent TGF-β has been shown to require numerous actors, such as various proteases and/or cell-surface receptors (such as RGD-dependent integrins), whose involvements are context- and tissue-dependent (30). Among the ECM molecules, TSP-1 was the first glycoprotein shown to activate latent TGF-β (51). TSP-1 is a trimeric protein which interacts non-covalently with latent TGF-β. The activation process involves the disruption of these non-covalent binding between the mature TGF-β cytokine and its LAP pro-domain through competing interactions between discrete sequences in TSP-1 and both components of the latent TGF-β complex (56). Firstly, the KRFK sequence located at the beginning of the second Thrombospondin type 1 repeat (TSR) can efficiently replace the RKPK sequence in mature TGF-β to interact with the LSKL sequence in LAP (bold sequences in **Figure S4**). This LSKL sequence is located in the first α-helix, just upstream the latency lasso (**Figure S4A**). Secondly, a repeated WXXW motif (where X is any amino acid residue) within TSP-1 has been shown to interact with the VLAL sequence in mature TGF-β (**Figure S4B**). It is assumed that these combined interactions subsequently induce a conformational change in the SLC and lead to the exposure of TGF-β, which otherwise remains buried within the straightjacket. Like TSP-1, TN-X is a trimeric glycoprotein, whose FBG-like domain interacts non-covalently with the SLC. After having excluded the involvement of proteases and RGD-dependent integrins, we deduced that latent TGF-β activation by TN-X most-likely occurred through a conformational change within the SLC (28). Even if a deeper analysis of the involved molecular mechanism is required, it is tempting to speculate that FBG-like domain of TN-C, TN-W and TN-R might also induce a conformational switch in this complex, thus leading to the exposure of mature TGF-β and its presentation to cell-surface receptors. Interestingly, like TSP-1, the FBG-like domain of the four Tenascins contains a conserved WXXW motif located in the loop 9, whose amino acids have been predicted to interact with the mature TGF-β1 (*, **Figure S3B**). As, from *in silico* analyses, the VLAL sequence in the bioactive TGF-β1 is unlikely to bind to the FBG-like domains (**Figure S4B**), the involvement of this tryptophan-rich motif will have to be confirmed experimentally following site-directed mutagenesis in the FBG globes. We also previously identified that α11β1 integrin was required for TN-X-mediated latent TGF-β activation (28). The exact role played by α11β1 integrin in latent TGF-β activation is still not clear. This cell-surface receptor might serve as a docking site for the FBG–LAP–TGF-β complex at the membrane, or might have an active role in the activation process by assisting the FBG-X domain to trigger the conformational change of the latent complex. Consequently, we cannot exclude the involvement of cell-surface receptor(s) for the activation of latent TGF-β1 by FBG-C, FBG-W and FBG-R. This question needs to be answered using RNA interference or neutralizing antibody approach, starting with αvβ3 integrin, a cell-surface receptor for the TN-C FBG-like domain (57). Nevertheless, this screening is challenged by the absence of identified cell-surface receptor for the FBG-like domain of TN-W and TN-R. Herein, by showing that latent TGF-β activation is a hallmark of the Tenascin family, we provide new evidences that these ECM glycoproteins are able to regulate cytokine activity and cellular signaling. Whether FBG-R retains the ability to regulate latent TGF-β1 activation within the full-length protein is an open question that will have to be answered in the future.

Tenascins are complex glycoproteins that appeared early in the chordate lineage and evolved into four conserved members in Coelacanth and Tetrapod (58). This degree of conservation suggests a crucial function in all Vertebrates. Because of its location at the very C-terminal end of Tenascin, the FBG-like globe is supposed to be more prone to be lost during evolution. However, this domain has been conserved, indicating the presence of a selective pressure to maintain it (59). This hypothesis becomes more and more attractive since the FBG knob function has been better characterized, although still underestimated. FBG-like domain function probably involves interactions with cell-surface receptors, other ECM components or signaling molecules. For instance, the FBG-like domain of TN-C, TN-W and TN-R, but not of TN-X, have been shown to interact with Toll-like receptor 4 (TLR4) to drive inflammatory cytokine synthesis *in vitro* and *in vivo* (60). Three distinct sites within the FBG-C domain contribute to TLR4 activation: (*i*) a cationic ridge made up of residues from loops 5–7, close to which sits (*ii*) a triad of hydrophobic/polar residues from loop 7 and (*iii*) a C-terminal cationic tail in loop 10. Whereas the cationic tail and the hydrophobic/polar residues seem non-essential; the cationic ridge is the dominant inflammatory epitope. To determine whether the binding of the LAP–TGF-β complex to the FBG-C domain might interfere with its ability to interact with TLR4, we highlighted key residues involved in TLR4 interaction in our molecular model (**Figure S7**). Latent TGF-β is predicted to lie on FBG-C in an opposite region from the three TLR4-binding sites. Although this has to be confirmed functionally, this observation suggests that the interaction of FBG-C with the latent TGF-β might not interfere with its ability to activate TLR4.

TGF-β has a dual role during carcinogenesis. Indeed, in normal or pre-malignant epithelial cells, it exerts tumor-suppressive activities by triggering apoptosis and cytostasis, thereby preventing malignant transformation (47, 48). In contrast, at later stages of tumor progression, TGF-β acts more as an oncogene by inducing EMT and immune evasion, thus promoting cancer cell invasion, dissemination and metastatic colonization. Interestingly, TN-X and TN-C/TN-W have opposite expression patterns in healthy and tumor tissues. Whereas TN-X is constitutively present in most adult connective tissues, we recently demonstrated a marked reduction of TN-X in the six most prevalent and lethal cancers worldwide (27). On the opposite, TN-C and TN-W are barely detectable in adult healthy tissues, but are often *de novo* expressed in cancers (23, 61), in which their ability to promote proliferation, migration and invasiveness have been extensively studied in the past decade (18, 22). More importantly, TN-C has been recently shown to promote EMT through activation of TGF-β canonical signaling pathway in breast cancer cells established using a mouse mammary tumor virus (MMTV) model (62). Up to date, molecular mechanisms underlying these processes are still not elucidated. Here, we show that the FBG-like domains of the four Tenascins induce cytostatic and EMT response in normal murine mammary gland epithelial cells *in vitro*. Based on opposite Tenascin expression patterns, it is tempting to speculate that TN-X might exert a TGF-β-dependent growth arrest in normal tissues or early malignant cells, whereas TN-C and TN-W induce latent TGF-β activation to trigger EMT at later stages of malignant transformation. Thus, this shared mechanism, at two different stages of cancer development, may have opposite impact on tumor progression. Strikingly, TN-C and TN-W have also been described as TGF-β responsive genes, that could lead to a positive retro-control loop (63, 64). Consequently, TN-C and TN-W, by their ability to activate latent TGF-β, can promote their own expression, resulting in a sustained TGF-β activation over time. In well-established tumors, this constant source of activated TGF-β may promotes tumor progression, increasing cancer invasiveness, aggressiveness and lethality. Indeed, even if Sun *et al.* also observed an increased TGF-β1 secretion in TN-C-stimulated murine breast cancer cells (62), this attractive hypothesis will require further investigation using established *in vivo* models.

Finally, TGF-β exerts a central role in tumor immune evasion (30). Indeed, this cytokine is produced by cancer cells but also by other cell types in the tumor microenvironment, including activated fibroblasts, macrophages, platelets and regulatory T cells (Tregs). High level of active TGF-β blocks naive T cell differentiation toward a Th1 effector phenotype, promotes their conversion toward the Treg subset and abolishes antigen-presenting functions of dendritic cells. Interestingly, the involvement of TN-C in the regulation of anti-tumor immunity has recently gained more attention. Tumor-initiating cells arising from prostate intraepithelial neoplasia or glioblastoma have been shown to secrete TN-C to protect themselves from immune surveillance, through an α5β1 or αvβ6 integrin-dependent inhibition of T cell activation and proliferation (65, 66). More recently, TN-C has also been shown to favor an immune-suppressive tumor microenvironment in oral squamous cell carcinoma, through the induction of a CCR7 signaling in dendritic cells, thus promoting the recruitment of T regulatory cells and the expression of anti-inflammatory cytokines (67). Knowing the role of TGF-β in tumor immune evasion, it would be relevant to investigate whether the immune-suppressive functions of TN-C (and maybe TN-W) might also depend on its ability to regulate TGF-β activity within these tumor microenvironments.

Considering the pleiotropic role of TGF-β in numerous physiological and pathological contexts, our data showing that latent TGF-β activation is a hallmark of the TN family shed light on a novel function for this ECM proteins in the regulation of tissue homeostasis and during pathological dysregulations.

## Data Availability Statement

The raw data supporting the conclusions of this article will be made available by the authors, without undue reservation.

## Author Contributions

AA: Conceptualization, Methodology, Investigation, Formal analysis; Writing – Original draft. PM-G: Validation, Writing – Review and Editing. LB: Software, Formal Analysis. SA: Software, Formal Analysis. RT: Software, Formal analysis, Writing – Review and Editing. SL: Writing – Review and Editing. LP: Writing – Review and Editing. LA: Writing – Review and Editing. BV: Funding acquisition. CM: Investigation, Formal analysis; Writing – Review & Editing. EL: Writing – Review and Editing. UV: Conceptualization, Writing – Original draft, Supervision, Funding Acquisition. All authors contributed to the article and approved the submitted version.

## Funding

This work was supported by the Centre National de la Recherche Scientifique (CNRS), the Ligue Nationale Contre le Cancer, Comité du Rhône (to UV), Comité de l’Allier (to UV) and Comité de la Saône-et-Loire (to BV), the Fondation ARC pour la Recherche sur le Cancer” (PJA 20141201790) (to UV), the French Association of Ehlers-Danlos Syndromes (AFSED) (to UV), and fellowships from the Ministère de l’Enseignement Supérieur, de la Recherche et de l’Innovation (MESRI) of France (LP) and from the Ecole Normale Supérieure de Lyon (AA and SL). The authors declare no competing financial interests.

## Conflict of Interest

The authors declare that the research was conducted in the absence of any commercial or financial relationships that could be construed as a potential conflict of interest.
