# Supplementary material for: Latent TGF-β Activation Is a Hallmark of the Tenascin Family
Source: Front Immunol. 2021 May 13;12:613438. doi: 10.3389/fimmu.2021.613438 (PMC8155481; doi:10.3389/fimmu.2021.613438)

Latent TGF-β activation is a hallmark of the Tenascin family

Alexandre AUBERT[1], Perrine MERCIER-GOUY[1], Stéphanie AGUERO[1], Laurent BERTHIER[1], Sophie LIOT[1], Laura PRIGENT[1], Lindsay B. ALCARAZ[2], Bernard VERRIER[1], Raphaël TERREUX[1], Catherine MOALI[1], Elise LAMBERT[1], Ulrich VALCOURT[1]*

[1]Laboratoire de Biologie Tissulaire et Ingénierie Thérapeutique (LBTI), UMR CNRS 5305, Université Lyon 1, Institut de Biologie et Chimie des Protéines, 7, passage du Vercors, F-69367 Lyon Cedex 07, France.

[2]Institut de Recherche en Cancérologie de Montpellier (IRCM), INSERM U1194, Université de Montpellier, Institut du Cancer de Montpellier (ICM), F-34298 Montpellier, France.

* ***Correspondence:*** Ulrich Valcourt (ulrich.valcourt@ibcp.fr)

Supplementary Materials and methods

Surface Plasmon Resonance (SPR) analyses

The interaction between recombinant human Latent TGF-β1 (LTGF-β1) (R&D Systems) or mature TGF-β1 (Peprotech) and the four FBG-like domains or purified TSP-1 was studied by surface plasmon resonance (SPR) as described in the main '*Materials and Methods*' section. TSP-1 was purified from human platelets as previously described (1). LTGF-β1 was immobilized on Series S CM5 sensorchips using amine coupling chemistry at pH 5.85 (in 10 mM sodium acetate).

Quanti-Blue™ SEAP reporter assay

HEK-Blue™ mTLR4 cells were obtained from InvivoGen and cultured in complete Dulbecco modified Eagle's medium (DMEM, Gibco) containing 10% (v/v) heat-inactivated Fetal Bovine Serum (FBS, Gibco), 1% (v/v) Penicillin-Streptomycin (5000 U/mL, Gibco) and HEK-Blue™ selection antibiotic cocktail (InvivoGen), as recommended by the manufacturer. 20,000 HEK-Blue™ mTLR4 cells were seeded in 96-well plates and stimulated overnight with different concentrations of LPS (from *E. coli* 055:B5; Invivogen). The next day, relative NF-κB activity was determined by measuring the SEAP activity accumulated in culture media (20 µL) using QUANTI-Blue™ (InvivoGen) according to the manufacturer's instructions. Absorbance was measured by spectrophotometry at 655nm on Tecan i-control Infinite M1000 (Tecan Group).

Quantitative real-time RT-PCR

50,000 NMuMG cells was seeded onto 6-well (or 12-well) plates coated or not (PBS) with 999 pmol/cm^2 (30 µg/cm^2) of purified recombinant human FBG-like domains and cultured in complete medium for 96h (or 72h). Total RNA was extracted from NMuMG cells with the RNeasy Plus Mini kit (QIAGEN) according to the manufacturer's instructions. Reverse transcription was performed with 1 µg RNA and 0,5 µg oligo-dT$_{12\text{-}18}$ primer (Thermo Scientific) in the presence of 200 U of SuperScript II reverse transcriptase (Thermo Scientific) and 40 U of RNaseOUT (Thermo Scientific). The cDNA was treated with 1 U of *E. coli* RNase H (Roche). Diluted cDNA (1:10) was analyzed by real-time quantitative PCR using FastStart Universal SYBR Green Master (Roche) in a Rotor-Gene Q system (QIAGEN) with specific primers (2). Results are expressed as relative values normalized with the reference gene *Gapdh* and quantified by the $2^{-\Delta\Delta Ct}$ method. The basal condition was set at 1 and expression data are presented as bar plots of mean values ± SD. Representative experiments are shown.

Data and statistical analysis

For quantitative real-time PCR experiments, statistical analyses were performed using paired Student *t*-test. Each condition was compared to the control condition (N-C) or its control IgG counterpart. p values < 0.05 were considered as statistically significant (p values were indicated in the figure legends).

References

1. Anastasi C, Rousselle P, Talantikite M, Tessier A, Cluzel C, Bachmann A, Mariano N, Dussoyer M, Alcaraz LB, Fortin L, et al. BMP-1 disrupts cell adhesion and enhances TGF-β activation through cleavage of the matricellular protein thrombospondin-1. *Sci Signal* (2020) **13**: aba3880.

2. Alcaraz LB, Exposito J-Y, Chuvin N, Pommier RM, Cluzel C, Martel S, Sentis S, Bartholin L, Lethias C, Valcourt U. Tenascin-X promotes epithelial-to-mesenchymal transition by activating latent TGF-β. *J Cell Biol* (2014) **205**:409–428.

3. Shi M, Zhu J, Wang R, Chen X, Mi L, Walz T, Springer TA. Latent TGF-β structure and activation. *Nature* (2011) **474**:343–349.

Supplementary Table I. Kinetic and dissociation constants derived from SPR analysis. See legend of Figure 3 for experimental conditions. Best fits for the interactions of TGF-β with hFBG-X and hFBG-W were obtained with the bivalent analyte model. *NA* = not applicable.

	k_{a1} ($M^{-1}s^{-1}$)	k_{d1} (s^{-1})	**K_{D1} (μM)**	k_{a2} ($RU^{-1}s^{-1}$)	k_{d2} (s^{-1})	Chi^2 (RU^2)
hFBG-X	$4.828\ 10^4$	0.09395	**1.95**	$8.052\ 10^{-6}$	0.001477	5.20
hFBG-C	*NA*	*NA*	*NA*	*NA*	*NA*	*NA*
hFBG-W	6862	0.08781	**12.80**	$1.596\ 10^{-6}$	0.001025	4.27
hFBG-R	*NA*	*NA*	*NA*	*NA*	*NA*	*NA*

Supplementary Figure 1. The recombinant FBG-like domain of Tenascin-W produced in mammalian cells is cleaved at its C-terminal cationic sequence $Arg^{1,217}$-Lys-Lys-$Arg^{1,220}$ (RKKR). Wild-type (FBG-W^{RKKR}) and mutated (FBG-W^{RKKA}) FBG-like domains of TN-W were produced in mammalian cells and purified as described in the “Materials and Methods” section. (Upper) purified recombinant proteins (2 μg each) were analyzed by SDS-PAGE (15% acrylamide gels under reducing conditions) coupled with Coomassie blue staining. MM, molecular mass markers. (Lower) Western blot analysis indicating the presence of the histidine tag on the purified recombinant FBG-W^{RKKR} and FBG-W^{RKKA} proteins.

Supplementary Figure 2. Recombinant mature TGF-β1, but not latent TGF-β1 (LTGF-β1) forms a complex with FBG-X. (A) Recombinant human LTGF-β1 was immobilized (2856 RU) and then FBG-X (5.8 μM), FBG-C (5.3 μM), FBG-W (8.6 μM) and FBG-R (8.6 μM) were injected. (B) Recombinant human LTGF-β1 was immobilized (2856 RU), then FBG-X (2 μM) and TSP-1 (500 nM) were injected. (A-B) Sensorgrams (relative response in RU after background subtraction *versus* times in sec) solely reflect “bulk” effect but not a physical interaction. (C) Recombinant human FBG-X was immobilized (1179 RU) and LTGF-β1 (100 and 315 nM) and mature TGF-β1 (100 and 500 nM) were injected. Sensorgrams indicate that mature TGF-β1, but not LTGF-β1, forms a complex with FBG-X.

Supplementary Figure 3. Molecular models predicting the interaction of pro-TGF-β1 with the FBG-like domain of Tenascins. (A) Homology models showing the predicted docking of the human FBG-X or FBG-R domain to pro-TGF-β1. The dimeric LAP(β1) pro-domain are shown in green, the TGF-β1 dimers in orange and the FBG-like domains in purple. (B) Sequence alignment of the four human FBG-like domains for which the amino acid residues that are predicted to interact with pro-TGF-β1 are highlighted in yellow. The conserved WXXW motif (where X is any amino acid residue) in loop 9 is indicated by asterisks (*).

Supplementary Figure 4. Amino acid residues of pro-TGF-β1 that are predicted to interact with each FBG-like domain. Amino acid sequences of human LAP(β1) pro-domain (Uniprot: P01137; amino acids: 30-278) (A) and human mature TGF-β1 (Uniprot: P01137; amino acids: 279-390) (B) for which the residues that are predicted to interact with each FBG-like domain of TNs are highlighted in yellow. The secondary structures of LAP(β1) pro-domain as well as

the sequences involved in TGF-β latency (red) are also indicated (from (3)). Cysteine^{355} is indicated in red.

Supplementary Figure 5. Lipopolysaccharides (LPS) and LPS-associated molecules do not induce Smad2 phosphorylation in NMuMG cells. (A) HEK-Blue mTLR4 cells were either left unstimulated (−) or stimulated with increasing concentrations of LPS for 16h and NF-kB activation was measured using QUANTI-Blue. Data are shown as mean ± SD from a representative experiment. (B) NMuMG cells were stimulated or not (-) for 1h with increasing concentrations of LPS (1, 10 and 100 ng/mL) or TGF-β1 (1 and 5 ng/mL) and the level of phosphorylated Smad2, total Smad2/3 and GAPDH were determined by immunoblotting. Fold changes of P-Smad2 to total Smad2 or to GAPDH levels are also indicated. (C) Relative Luciferase activity of NMuMG cells transiently transfected with the Smad-responsive $(CAGA)_{12}$-Luc reporter construct and treated for 16h with increasing concentrations of LPS (1, 10 and 100 ng/mL) or TGF-β1 (5 ng/mL). Data are shown as mean ± SEM from three independent experiments.

Supplementary Figure 6. Recombinant FBG-like of the four Tenascins differentially regulate EMT in mammary epithelial cells. (A) Quantitative real-time RT-PCR analysis of known EMT markers and regulators in NMuMG cells treated for 96h onto non-coated (N-C) dishes or dishes coated with equimolar quantity of FBG-like domains (333 pmol/cm^2), or stimulated with soluble active TGF-β1 (5 ng/mL). Error bars are means ± SD of a representative experiment. *, **, *** and **** respectively correspond to $p < 0.05$, $p < 0.01$, $p < 0.001$ and $p < 0.0001$ compared to N-C condition. (B) Quantitative real-time RT-PCR analysis of known EMT markers and regulators in NMuMG cells treated for 72h onto non-coated (N-C) dishes or dishes coated with equimolar quantity of FBG-X or FBG-C domains (333 pmol/cm^2), or stimulated with soluble active TGF-β1 (5 ng/mL), in the presence of anti-TGF-β1/2/3 antibody or isotype-matched control IgG (5 μg/mL). CUB1CUB2 (C1C2) fragment protein was added as a negative control (333 pmol/cm^2). Graphs show means ± SD of a representative experiment. *, **, *** and **** respectively correspond to $p < 0.05$, $p < 0.01$, $p < 0.001$ and $p < 0.0001$ compared to N-C condition. $^{\#}$, $^{\#\#}$, $^{\#\#\#}$ and $^{\#\#\#\#}$ respectively correspond to $p < 0.05$, $p < 0.01$, $p < 0.001$ and $p < 0.0001$ versus their control IgG-treated counterpart.

Supplementary Figure 7. The FBG-like domain of TN-C interacts with latent TGF-β1 and Toll-like Receptor 4 (TLR4) through distinct binding interfaces. Molecular model of the pro-TGF-β1–FBG-C complex, in which the amino acid residues involved in TLR4 binding are highlighted. The pro-TGF-β1 is depicted in orange and the FBG-like domain of TN-C (FBG-C) is showed in gray. The positively charged residues located in the cationic ridge and tail are colored red, whereas the triad of polar and hydrophobic residues conserved in FBG-C, FBG-R, and FBG-W (but absent in FBG-X) are colored in green.

Supp. Fig 1 – AUBERT *et al.*

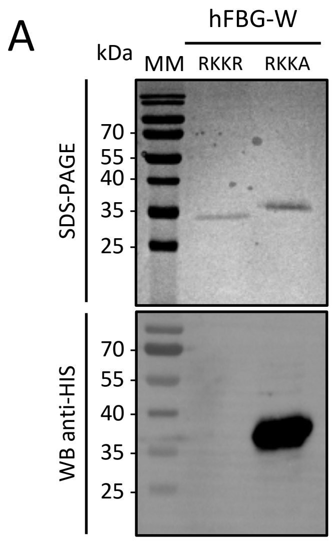

Supp. Fig 2 – AUBERT *et al.*

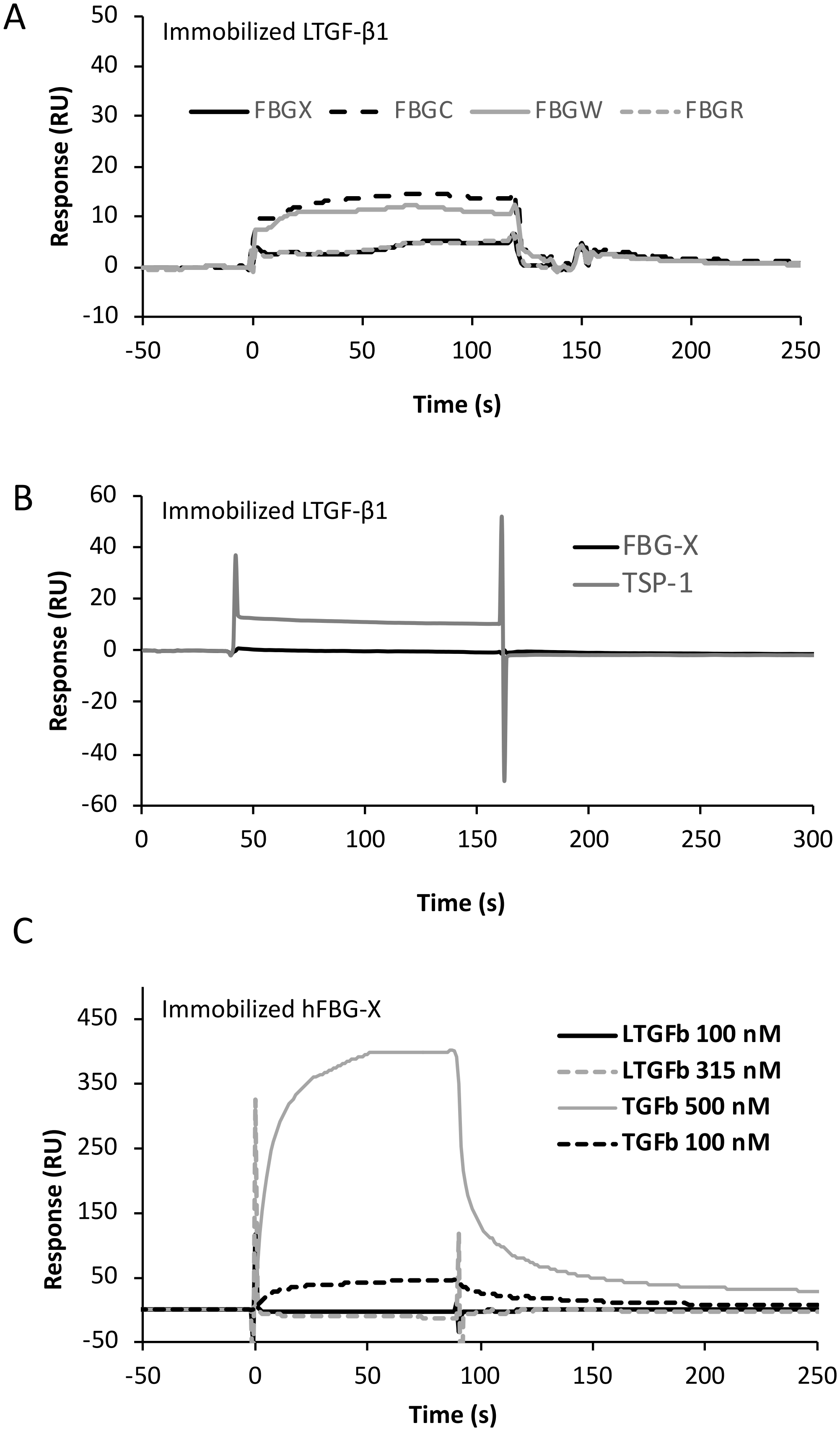

Supp. Fig 3 – AUBERT *et al.*

A

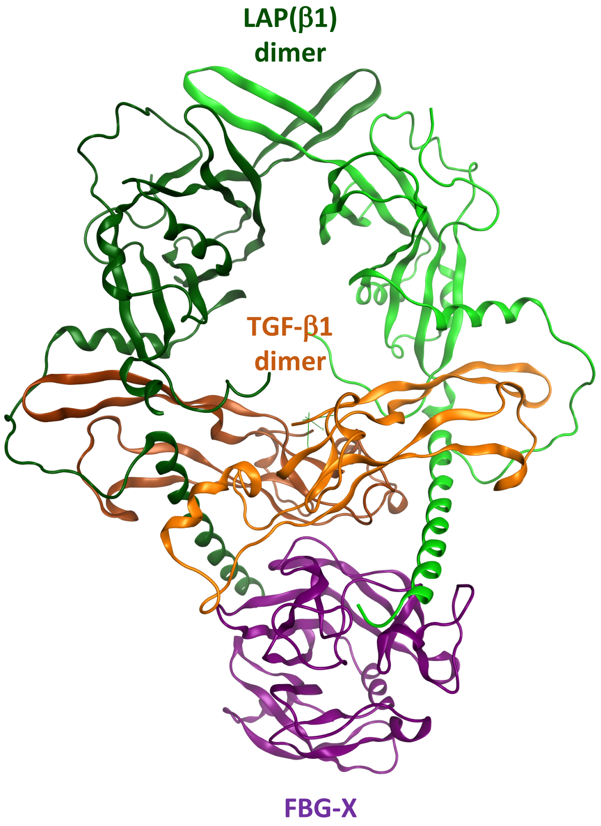

B

```
                  α helix   Loop 1  β sheet  Loop 2     β sheet           β sheet
FBG-X     GGLRIPFPRDCGEEMQNGAGASRTSTIFLNGNRERPLNVFCDMETDGGGWLVFQRRMDGQ
FBG-C     IGLLYPFPKDCSQAMLNGDTTSGLYTIYLNGDKAQALEVFCDMTSDGGGWIVFLRRKNGR
FBG-W     VGARFPHPSDCSQVQQNSNAASGLYTIYLHGDASRPLQVYCDMETDGGGWIVFQRRNTGQ
FBG-R     GGRVFPHPQDCAQHLMNGDTLSGVYPIFLNGELSQKLQVYCDMTTDGGGWIVFQRRQNGQ

           Loop 3   α helix   Loop 4     α helix     β sheet        β sheet
FBG-X     TDFWRDWEDYAHGFGNISGEFWLGNEALHSLTQ--AGDYSMRVDLRAGDEAVFAQYDSFH
FBG-C     ENFYQNWKAYAAGFGDRREEFWLGLDNLNKITA--QGQYELRVDLRDHGETAFAVYDKFS
FBG-W     LDFFKRWRSYVEGFGDPMKEFWLGLDKLHNLTTGTPARYEVRVDLQTANESAYAIYDFFQ
FBG-R     TDFFRKWADYRVGFGNVEDEFWLGLDNIHRITS--QGRYELRVDMRDGQEAAFASYDRFS

             Loop 5         Loop 6          Loop 7      α helix   Loop 8
FBG-X     VDSAAEYYRLHLEGYHGTAGDSMSYHSGSVFSARDRDPNSLLISCAVSYRGAWWYRNCHY
FBG-C     VGDAKTRYKLKVEGYSGTAGDSMAYHNGRSFSTFDKDTDSAITNCALSYKGAFWYRNCHR
FBG-W     VASSKERYKLTVGKYRGTAGDALTYHNGWKFTTFDRDNDIALSNCALTHHGGWWYKNCHL
FBG-R     VEDSRNLYKLRIGSYNGTAGDSLSYHQGRPFSTEDRDNDVAVTNCAMSYKGAWWYKNCHR

                  Loop 9            β sheet  α helix  Loop 10
FBG-X     ANLNGLYGSTVDHQGVSWYHWKGFEFSVPFTEMKLRP--RNFRSPAGGG-----------
FBG-C     VNLMGRYGDNNHSQGVNWFHWKGHEHSIQFAEMKLRP--SNFRNLEGRRKRA--------
FBG-W     ANPNGRYGETKHSEGVNWEPWKGHEFSIPYVELKIRPHGYSREPVLGRKKRTLRGRLRTF
FBG-R     TNLNGKYGESRHSQGINWYHWKGHEFSIPFVEMKMRP--YNHRLMAGRKRQSLQ-----F
                            ****
```

Supp. Fig 4 – AUBERT *et al.*

A Human LAP(β1) (Uniprot: P01137; aminoacids: 30-278)

α helix 1 *Latency lasso* *α helix 2*

FBG-X LSTCKTIDMELVKRKRIEAIRGQI**LSKL**RLASPPSQGEVPPGPLPEAVLALYNSTRDRVA
FBG-C LSTCKTIDMELVKRKRIEAIRGQI**LSKL**RLASPPSQGEVPPGPLPEAVLALYNSTRDRVA
FBG-W LSTCKTIDMELVKRKRIEAIRGQI**LSKL**RLASPPSQGEVPPGPLPEAVLALYNSTRDRVA
FBG-R LSTCKTIDMELVKRKRIEAIRGQI**LSKL**RLASPPSQGEVPPGPLPEAVLALYNSTRDRVA

Fastener *β Sheet 1* *β Sheet 2* *α helix 3*

FBG-X GESAEPEPEPEADYYAKEVTRVLMVETHNEIYDKFKQSTHSIYMFFNTSELREAVPEPVL
FBG-C GESAEPEPEPEADYYAKEVTRVLMVETHNEIYDKFKQSTHSIYMFFNTSELREAVPEPVL
FBG-W GESAEPEPEPEADYYAKEVTRVLMVETHNEIYDKFKQSTHSIYMFFNTSELREAVPEPVL
FBG-R GESAEPEPEPEADYYAKEVTRVLMVETHNEIYDKFKQSTHSIYMFFNTSELREAVPEPVL

β Sheet 3 *β Sheet 4* *β Sheet 5* *β Sheet 6* *α helix 4*

FBG-X LSRAELRLLRLKLKVEQHVELYQKYSNNSWRYLSNRLLAPSDSPEWLSFDVTGVVRQWLS
FBG-C LSRAELRLLRLKLKVEQHVELYQKYSNNSWRYLSNRLLAPSDSPEWLSFDVTGVVRQWLS
FBG-W LSRAELRLLRLKLKVEQHVELYQKYSNNSWRYLSNRLLAPSDSPEWLSFDVTGVVRQWLS
FBG-R LSRAELRLLRLKLKVEQHVELYQKYSNNSWRYLSNRLLAPSDSPEWLSFDVTGVVRQWLS

β Sheet 7 *β Sheet 8* *β Sheet 9* *β Sheet 10* *α helix 5*

FBG-X RGGEIEGFRLSAHCSCDSRDNTLQVDINGFTTGRRGDLATIHGMNRPFLLLMATPLERAQ
FBG-C RGGEIEGFRLSAHCSCDSRDNTLQVDINGFTTGRRGDLATIHGMNRPFLLLMATPLERAQ
FBG-W RGGEIEGFRLSAHCSCDSRDNTLQVDINGFTTGRRGDLATIHGMNRPFLLLMATPLERAQ
FBG-R RGGEIEGFRLSAHCSCDSRDNTLQVDINGFTTGRRGDLATIHGMNRPFLLLMATPLERAQ

FBC-C HLQSSRHRR
FBG-W HLQSSRHRR
FBG-R HLQSSRHRR

B Human mature TGF-β1 (Uniprot: P01137; aminoacids: 279-390)

FBG-X ALDTNYCFSSTEKNCCVRQLYIDFRKDLGWKWIHEPKGYHANFCLGPCPYIWSLDTQYSK
FBG-C ALDTNYCFSSTEKNCCVRQLYIDFRKDLGWKWIHEPKGYHANFCLGPCPYIWSLDTQYSK
FBG-W ALDTNYCFSSTEKNCCVRQLYIDFRKDLGWKWIHEPKGYHANFCLGPCPYIWSLDTQYSK
FBG-R ALDTNYCFSSTEKNCCVRQLYIDFRKDLGWKWIHEPKGYHANFCLGPCPYIWSLDTQYSK

FBG-X **VLAL**YNQHNPGASAAPCCVPQALEPLPIVYYVG**RKPK**VEQLSNMIVRSCKCS
FBG-C **VLAL**YNQHNPGASAAPCCVPQALEPLPIVYYVG**RKPK**VEQLSNMIVRSCKCS
FBG-W **VLAL**YNQHNPGASAAPCCVPQALEPLPIVYYVG**RKPK**VEQLSNMIVRSCKCS
FBG-R **VLAL**YNQHNPGASAAPCCVPQALEPLPIVYYVG**RKPK**VEQLSNMIVRSCKCS

Supp. Fig 5 – AUBERT *et al.*

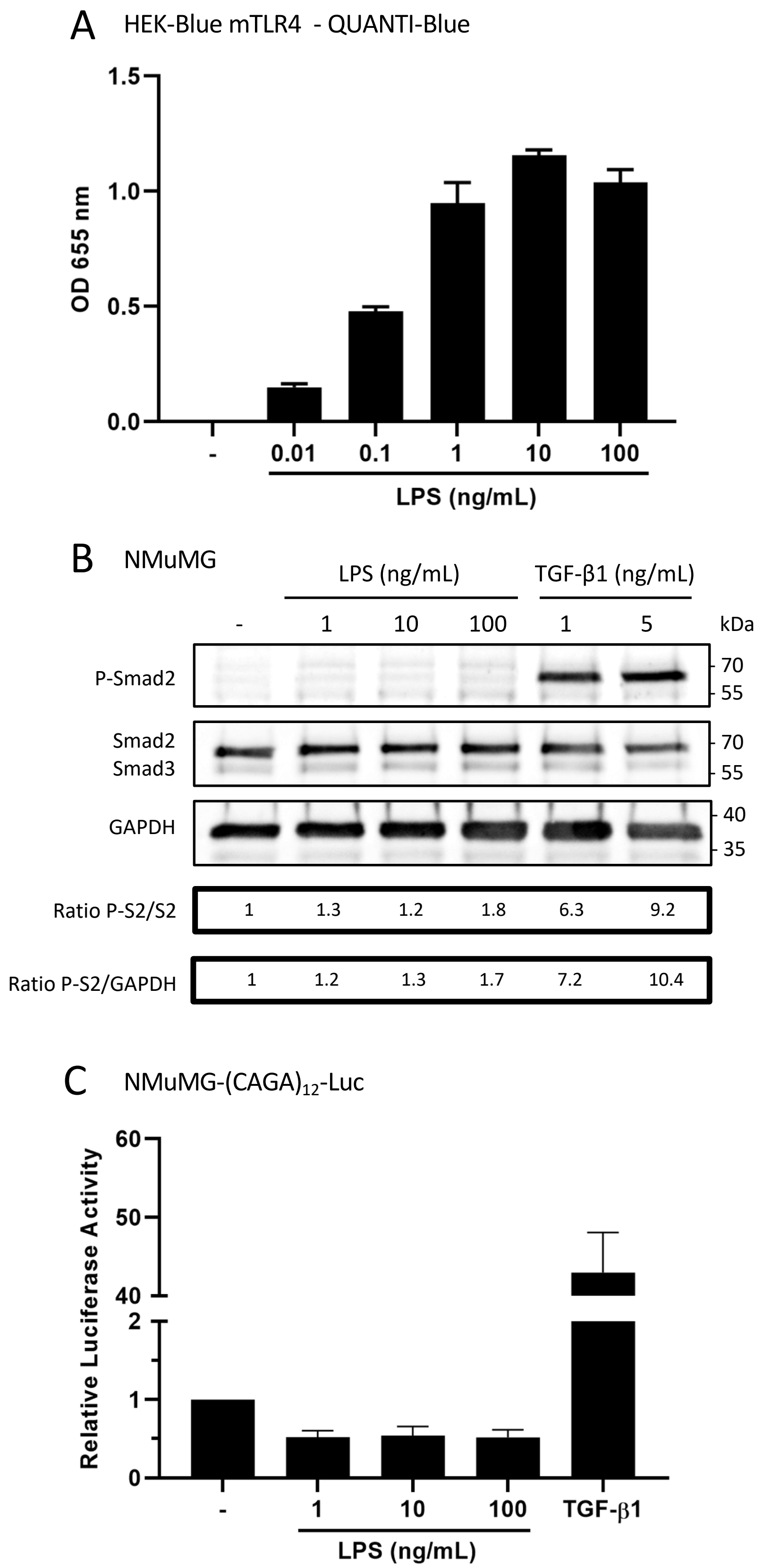

Supp. Fig 6 – AUBERT *et al.*

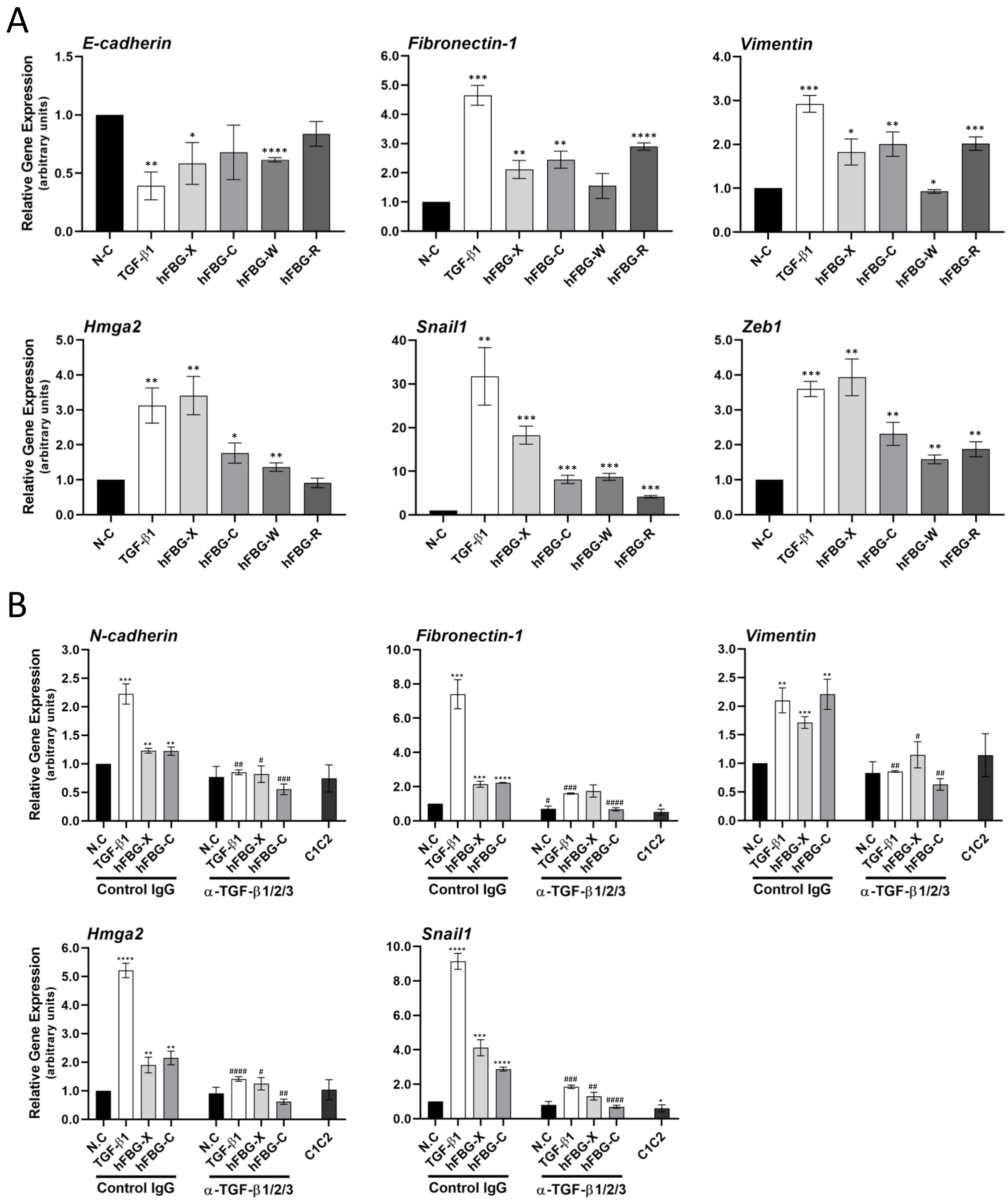

Supp. Fig 7 – AUBERT *et al.*

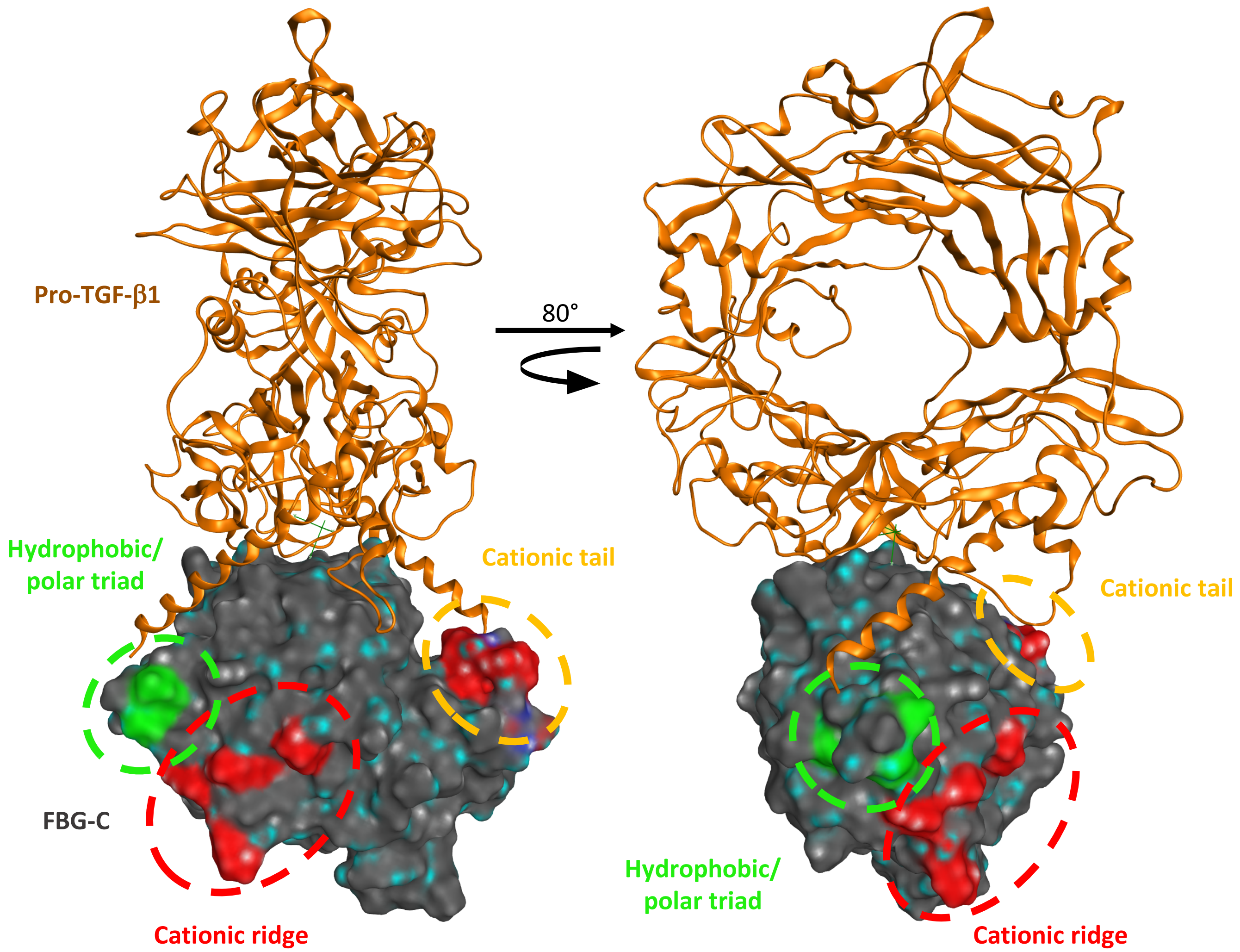

Supplement: Supplementary file 1 [file DataSheet_1.pdf]
